# Distinct cognitive changes in male patients with obstructive sleep apnoea without co-morbidities

**DOI:** 10.3389/frsle.2023.1097946

**Published:** 2023-04-06

**Authors:** Valentina Gnoni, Michel Mesquita, David O'Regan, Alessio Delogu, Ivan Chakalov, Andrea Antal, Allan H. Young, Romola S. Bucks, Melinda L. Jackson, Ivana Rosenzweig

**Affiliations:** ^1^Sleep and Brain Plasticity Centre, Department of Neuroimaging, Institute of Psychiatry, Psychology and Neuroscience, King's College London, London, United Kingdom; ^2^L&M Data Science Ltd., London, United Kingdom; ^3^Sleep Disorder Centre, Nuffield House, Guy's Hospital, London, United Kingdom; ^4^Faculty of Life Sciences and Medicine, King's College London, London, United Kingdom; ^5^Institute of Psychiatry, Psychology and Neuroscience, King's College London, London, United Kingdom; ^6^Department of Anesthesiology, University Medical Center Göttingen, Göttingen, Germany; ^7^Department of Neurology, University Medical Center Göttingen, Göttingen, Germany; ^8^Department of Psychological Medicine, Institute of Psychiatry, Psychology and Neuroscience, King's College London, London, United Kingdom; ^9^South London and Maudsley National Health Service (NHS) Foundation Trust, Bethlem Royal Hospital, Beckenham, United Kingdom; ^10^School of Psychological Science, University of Western Australia, Perth, WA, Australia; ^11^The Raine Study, School of Population and Global Health, University of Western Australia, Perth, WA, Australia; ^12^Turner Institute for Brain and Mental Health, School of Psychological Sciences, Monash University, Melbourne, VIC, Australia

**Keywords:** sleep, cognition, obstructive sleep apnea, comorbidity, social memory

## Abstract

**Introduction:**

Obstructive sleep apnoea (OSA) is a multisystem, debilitating, chronic disorder of breathing during sleep, resulting in a relatively consistent pattern of cognitive deficits. More recently, it has been argued that those cognitive deficits, especially in middle-aged patients, may be driven by cardiovascular and metabolic comorbidities, rather than by distinct OSA-processes, such as are for example ensuing nocturnal intermittent hypoxaemia, oxidative stress, neuroinflammation, and sleep fragmentation.

**Methods:**

Thus, we undertook to define cognitive performance in a group of 27 middle-aged male patients with untreated OSA, who had no concomitant comorbidities, compared with seven matched controls (AHI mean ± S.D.: 1.9 ± 1.4 events/h; mean age 34.0 ± 9.3 years; mean BMI 23.8 ± 2.3 kg/m^2^). Of the 27 patients, 16 had mild OSA (AHI mean ± S.D.:11.7 ± 4.0 events/h; mean age 42.6 ± 8.2 years; mean BMI 26.7 ± 4.1 kg/m^2^), and 11 severe OSA (AHI 41.8 ± 20.7 events/h; age: 46.9 ± 10.9 years, BMI: 28.0 ± 3.2 kg/m^2^).

**Results:**

In our patient cohort, we demonstrate poorer executive-functioning, visuospatial memory, and deficits in vigilance sustained attention, psychomotor and impulse control. Remarkably, we also report, for the first time, effects on social cognition in this group of male, middle-aged OSA patients.

**Conclusion:**

Our findings suggest that distinct, OSA-driven processes may be sufficient for cognitive changes to occur as early as in middle age, in otherwise healthy individuals.

## 1. Introduction

Obstructive sleep apnoea (OSA) is a multisystem, debilitating, chronic disorder of breathing during sleep, resulting in a relatively consistent pattern of cognitive deficits (Rosenzweig et al., 2015; Bucks et al., 2017), particularly in attention, executive function and episodic memory (Bucks et al., 2017). Moreover, there is a high prevalence of depression, anxiety and other psychiatric problems, which are only partially remediated by treatment (Rosenzweig et al., 2015; Bucks et al., 2017).

Cognitive functions traditionally comprise broad domains of attention and memory, as well as those of higher order cognitive skills such as planning, problem-solving, and mental flexibility (grouped together as executive function), visuospatial abilities, processing speed, and both expressive and receptive language (Esther Strauss, 2006; Bucks et al., 2017; Rosenzweig et al., 2022). Historically, a body of work has suggested significant impact of OSA on: attention and vigilance, long-term verbal and visual memory, expressive and receptive language (Bucks et al., 2013; Wallace and Bucks, 2013), and visuo-spatial and constructional abilities (Bucks et al., 2013). Similarly, deficits in the executive domain have also been demonstrated (Olaithe and Bucks, 2013; Bucks et al., 2017), with somewhat less uniform evidence for short-term memory deficits (Rosenzweig et al., 2022). However, cognitive domains are not unitary constructs, and only judiciously deconstructed analysis of their different sub-capacities and their vulnerabilities to a range of risks and protective factors specific to OSA can provide a more accurate appraisal of a patient's deficits (Rosenzweig et al., 2017, 2022).

Accordingly, OSA's bidirectional link to neurodegenerative disorders, including Alzheimer's disorder (AD), has similarly highlighted the importance of disentangling some of the major cognitive neuromechanisms at play (Ancoli-Israel et al., 2008; Cooke et al., 2009; Osorio et al., 2015; Bubu et al., 2022). However, the links between severity of OSA, historically indexed by apnea-hypopnea index (AHI) or respiratory disturbance index (RDI), or by indices of hypoxia severity, sleep fragmentation, or sleepiness (Pépin et al., 2005; Bucks et al., 2017), and the severity of the cognitive deficits observed, are far from being well understood (for more in-depth review please refer to Aloia et al., 2004; Olaithe and Bucks, 2013; Wallace and Bucks, 2013; Gagnon et al., 2014; Rosenzweig et al., 2015; Bucks et al., 2017). Similarly poorly understood, is the link between the timing of the hypoxia or arousal during each sleep cycle, and the severity or the cognitive phenotype (Rosenzweig et al., 2015).

Moreover, the link between cognition, OSA, and aging, has proven equally difficult to fully discern (Rosenzweig et al., 2022). For instance, aging is known to be independently linked with physiological changes that may predispose to OSA (Rosenzweig et al., 2022). It has been proposed that this may be in part due to changes in upper airway morphology that can lead to a reduction in upper airway dilator muscle function at sleep onset (Bucks et al., 2017), contributing to an age-related propensity for upper airway collapse in response to negative pressure (Kirkness et al., 2008) independent of body mass index (Eikermann et al., 2007; Rosenzweig et al., 2022). Against this background, it has been argued that some of these aging-associated processes may underlie the failure to find a consistent relationship between the severity of OSA and the risk of cognitive impairment (Bucks et al., 2017; Rosenzweig et al., 2022).

This evident polymorphic picture is further compounded by the fact that not everyone with OSA is cognitively impaired (Quan et al., 2006), with the individual's cognitive reserve (Alchanatis et al., 2005; Sforza et al., 2010; Bucks et al., 2013; Martin et al., 2015; Olaithe et al., 2015; Schembri et al., 2017) and their genetic risk (Cosentino et al., 2008; Nikodemova et al., 2013) for neurodegenerative decline (Bucks et al., 2017) possibly also playing an important role (Rosenzweig et al., 2022).

Taken together, it has been proposed that only middle-aged patients with OSA may demonstrate a consistent pattern of cognitive deficits, otherwise lacking in older patients (Bubu et al., 2020). Notably, it has also been argued that deficits are principally driven by common cardiovascular and metabolic comorbidities, rather than by distinct OSA-processes (Bubu et al., 2020). In further support of this, patients with OSA invariably present with already established comorbidities, such as overweight or obesity, sleepiness in passive situations or while driving, and are often affected by systemic hypertension, type 2 diabetes, and dyslipidemia (Levy et al., 2015), making it in most cases impossible to delineate the specific contribution of all associated risks to the resulting cognitive presentation (Bonsignore et al., 2019; Bubu et al., 2020; Rosenzweig et al., 2022).

Whether OSA itself, or these common comorbidities drive the cognitive effects has wide ranging clinical implications, and may impact future clinical guidelines with treatment of comorbidities taking precedence over treatment of the core determinants of neuropathological process in OSA, such as sleep fragmentation (Jordan et al., 2014), sleep disruption and blood gas abnormalities (Olaithe et al., 2018).

To this end, and in order to clarify whether, independent of concomitant metabolic or cardiovascular comorbidities, OSA-induced injury (Rosenzweig et al., 2015) may present with abnormal functional outcomes (Bubu et al., 2020; Rosenzweig et al., 2022), we undertook a proof of the concept study and set to define the cognitive pattern in a (rare) group of male, middle-aged patients with untreated OSA who present without comorbidities, compared to matched controls.

## 2. Methods

Preliminary analysis of cognitive parameters in patients with different OSA severities was undertaken as a part of the multimodal clinical study InCOSA (Clinical.Trials.gov, identifier: NCT02967536). All experimental protocols were approved by the U.K. Research Ethics Committee [Integrated Research Application System (IRAS): IRAS-Project-ID-170912; REC-REF16/L0/0893] and informed consent for study participation was obtained from all participants. Due to the nature of several investigations in the overarching multimodal clinical study, some of which are known to have significant sex and gender differences, only male participants were included. Thus, 27 male adult (35–70 years-old), non-obese [body-mass-index (BMI) < 30 kg/m^2^], mildly somnolent [Epworth Sleepiness Scale (ESS); 5 > ESS < 15] patients with no current or past co-morbidities, and no current or past alcohol or smoking history, with a *de novo* diagnosis of OSA according to ICSD criteria (American Academy of Sleep Medicine, 2014), and a group of healthy sex- and education-matched individuals were identified, as previously described (Gnoni et al., 2021) (Supplementary material). All methods were carried out in accordance with relevant UK and international guidelines and regulations.

All participants underwent a domiciliary respiratory testing *via* WatchPAT system (https://www.itamar-medical.com/), as previously described (Walter et al., 2023). Additionally, patients also underwent a video-polysomnography (vPSG) in the sleep center. Full night vPSG recordings were based on the international 10:20 system; for purposes of the PSG scoring, six EEG channels (i.e., F3, F4, C3, C4, O1, and O2) were referenced to the mastoid, and used along with electrooculography, submental-electromyography, respiratory inductance plethysmography, nasal pressure sensor, oronasal thermistor, pulse-oximeter, two-lead electrocardiogram, body position detector and synchronized audio-visual recording, as previously described (Rosenzweig et al., 2016). The scoring was carried out according to AASM rules (Berry et al., 2017), and as previously described (Rosenzweig et al., 2016). Eleven cognitive domains were pre-selected (Bucks et al., 2017), based on previous reports of OSA- and depression-related deficits, and assessed *via* 23 automated Cambridge-Neuropsychological-Test-Automated-Battery (CANTAB) tests.

### 2.1. Cambridge neuropsychological test automated battery

CANTAB is a highly sensitive, validated touchscreen-based cognitive assessment. In this study, 11 domains were tested with the following tests (for more in-depth explanations please refer to Supplementary material). *Reaction Time Task (RTT)* (Cognition, n.d.a) tests reaction time, movement time, and vigilance, which are associated with motor pathway and right anterior hemispheric functioning (Coull et al., 1998). *Spatial Working Memory (SWM)* tests (Cognition, n.d.b) the retention and manipulation of visuospatial data in non-verbal and visuospatial working memory (Cognition, n.d.b), which are associated with frontal lobe function. *Pattern Recognition Memory (PRM)* tests (Cognition, n.d.a) short-term visual memory in a two choice forced discrimination paradigm in both immediate and delayed conditions, which are associated with frontoparietal and posterior parietal function (Pessoa et al., 2002; Todd and Marois, 2004). *The Emotion Recognition Task (ERT)* (Cognition, n.d.c) assesses social cognition and emotion recognition (Glenthøj et al., 2019), which are associated with the limbic system, inferior frontal gyrus, parietal lobe, cingulate cortex and inferior and middle temporal lobe functioning (Keightley et al., 2011). Participants are shown a computer-generated face for 200 ms, after which the emotion displayed by the face must be selected from six options, i.e., sadness, happiness, fear, anger, disgust, and surprise. The outcome measures are the median reaction time and the total number of correct answers (Cognition, n.d.c). People with depression are likely to provide more negative ratings of emotional expression, reflecting the well-known negative bias seen in depression (Cognition, n.d.c). During testing, brief presentation encourages implicit processing, as opposed to conscious appraisal of the faces (Cognition, n.d.c). Conversely, in individuals at ultra-high risk of developing psychosis, longer emotion recognition latency, rather than lower accuracy has been demonstrated (Glenthøj et al., 2019). *The Attention Switching Task (AST)* (Cognition, n.d.d) tests executive functioning and cued attentional set-shifting, which are functions of the medial frontal structures and the anterior right hemisphere (Bench et al., 1993). *Spatial Span Memory (SSP)* (Cognition, n.d.d) assesses visuospatial working memory capacity which is associated with frontoparietal function (Jones and Berryhill, 2012; Ester et al., 2015). *The Paired Associates Learning (PAL)* tests (Cognition, n.d.e) episodic visuospatial memory and associative learning, which are predominantly functions of the temporal lobe. One *Touch Stockings of Cambridge (OTS)* (Cognition, n.d.f) tests spatial planning and working memory and it is a measure of dorsolateral prefrontal cortex function (Goldman-Rakic, 1995). *Delayed Matching to Sample* (DMS) (Cognition, n.d.g) assesses both simultaneous visual matching ability and short-term visual recognition memory, for non-verbalizable patterns, which is associated with medial temporal lobe function (Lavenex et al., 2002; Lee et al., 2005). The *Rapid Visual Information Processing (RVP)* (Cognition, n.d.g) is a measure of sustained attention, associated with frontoparietal function (Sarter et al., 2001). *Stop Signal Task (SST)* (Cognition, n.d.h) is a test of impulse control and response inhibition associated with prefrontal cortex function (Sarter et al., 2001).

### 2.2. Statistical analyses

Group differences were analyzed with 2-way ANCOVA, corrected for multiple comparisons using Bonferroni test with additional pairwise tests. Age and BMI are used as covariates in the ANCOVA model and are evaluated at the following values: Age = 41.59 years, BMI = 26.78 kg/m^2^. Differences in socio-demographic characteristics are evaluated with *t*-test for independent samples (continuous variables) and Fisher's exact test or Fisher-Freeman-Halton's exact test (categorical variables). All *P*-values below 0.05 were considered significant. MedCalc^®^ Statistical Software version 20.216 (MedCalc Software Ltd., Ostend, Belgium; https://www.medcalc.org; 2023) was used in statistical analysis and graphical presentations.

## 3. Results

Twenty-seven OSA patients and seven healthy controls (see Table 1) completed the CANTAB (Table 2). Of the 27 patients, 16 were diagnosed with mild OSA (AHI mean ± S.D.:11.7 ± 4.0 events/h; mean age 42.6 ± 8.2 years; mean BMI 26.7 ± 4.1 kg/m^2^), and 11 with severe OSA (AHI 41.8 ± 20.7 events/h; age: 46.9 ± 10.9 years, BMI: 28.0 ± 3.2 kg/m^2^), according to ICSD criteria (American Academy of Sleep Medicine, 2014).

**Table 1:** Socio-demographic and clinical characteristics.

		**HC**	**Mild OSA**	**Severe OSA**	**Control vs. Mild OSA**	**Control vs. Severe OSA**	**Mild OSA vs. Severe OSA**
		**(*****n*** = **7)**	**(*****n*** = **16)**	**(*****n*** = **11)**	***P***	***P***	***P***
Age (years)^a^	Mean (SD)	34.0 (9.3)	42.6 (8.2)	46.9 (10.9)	0.0380	0.0199	0.2484
BMI (kg/m^2^)^a^	Mean (SD)	23.8 (2.3)	26.7 (4.1)	28.0 (3.2)	0.0957	0.0094	0.4137
AHI^a^	Mean (SD)	1.9 (1.4)	11.7 (4.0)	41.8 (20.7)	<0.0001	0.0001	<0.0001
**Ethnicity** ^b^					0.6971	0.5769	0.1986
Caucasian	*n* (%)	5 (71.4)	13 (81.3)	7 (63.6)			
Asian	*n* (%)	2 (28.6)	2 (12.5)	1 (9.1)			
Indian	*n* (%)	0 (0.0)	1 (6.3)	0 (0.0)			
Afro-Caribbean	*n* (%)	0 (0.0)	0 (0.0)	2 (18.2)			
Middle east	*n* (%)	0 (0.0)	0 (0.0)	1 (9.1)			
**Smoking** ^b^					0.1243	1.0000	0.0535
Ex-smoker	*n* (%)	0 (0.0)	6 (37.5)	0 (0.0)			
Non-smoker	*n* (%)	7 (100.0)	10 (62.5)	11 (100.0)			
Smoker	*n* (%)	0 (0.0)	0 (0.0)	0 (0.0)			
**Education** ^b^					0.0735	0.2450	0.1614
Undergrade	*n* (%)	2 (28.6)	11 (68.8)	4 (36.4)			
Graduate	*n* (%)	5 (71.4)	3 (18.7)	6 (54.5)			
A level	*n* (%)	0 (0.0)	2 (12.5)	1 (9.1)			
**Age at leaving education** ^a^	Mean (SD)	25.6 (4.1)	22.5 (3.2)	26.3 (9.3)	0.0627	0.8535	0.2184
**Exercise regularly** ^c^					0.1243	0.2450	0.6924
No	*n* (%)	0 (0.0)	6 (37.5)	3 (27.3)			
Yes	*n* (%)	7 (100.0)	10 (62.5)	8 (72.7)			

a T-test for independent samples.

b Fisher-Freeman-Halton's exact test.

c Fisher's exact test.

**Table 2:** Estimated marginal means of analyzed clinical parameters controlled for the influence of age and BMI (two-way ANCOVA).

**Dependent variable**	**Group**											
	**Control** ***n*** = **7**				**Mild OSA** ***n*** = **16**				**Severe OSA** ***n*** = **11**			
	**Mean**	**SEM**	**95% CI**		**Mean**	**SEM**	**95% CI**		**Mean**	**SEM**	**95% CI**	
			**Lower**	**Upper**			**Lower**	**Upper**			**Lower**	**Upper**
ASTLSWMD	564.45	48.30	464.98	663.93	758.77	31.92	693.04	824.50	789.18	41.22	704.30	874.07
ASTLCMD	702.94	36.67	627.42	778.46	626.88	24.23	576.97	676.78	520.67	31.29	456.22	585.11
ERTOMDRT	1,939.65	195.30	1,537.41	2,341.88	1,398.66	129.05	1,132.88	1,664.44	1,133.05	166.66	789.80	1,476.29
ERTTH	48.92	3.96	40.76	57.09	57.49	2.62	52.10	62.89	64.89	3.38	57.92	71.85
PALTEA	14.76	4.22	6.06	23.46	14.63	2.79	8.88	20.37	9.24	3.60	1.82	16.66
PALFAMS	5.45	1.49	2.38	8.52	4.81	0.98	2.78	6.84	7.35	1.27	4.73	9.97
PRMPCI	99.78	3.15	93.30	106.25	91.98	2.08	87.70	96.26	96.58	2.68	91.05	102.11
PRMPCD	78.29	5.58	66.79	89.78	88.51	3.69	80.91	96.10	88.17	4.76	78.36	97.98
RTIFDMRT	391.42	12.82	365.02	417.82	384.78	8.47	367.34	402.23	349.17	10.94	326.65	371.70
RTIFMMT	266.90	13.47	239.17	294.64	240.85	8.90	222.52	259.17	197.59	11.49	173.92	221.25
SWMBE	23.81	4.60	14.33	33.28	19.05	3.04	12.79	25.31	14.58	3.93	6.49	22.66
SWMS	5.59	1.25	3.03	8.16	4.47	0.82	2.77	6.16	5.51	1.06	3.32	7.70
SSPSFSL	5.81	0.53	4.71	6.91	6.91	0.35	6.18	7.64	7.60	0.46	6.66	8.54
SSPRSL	5.91	0.57	4.73	7.09	6.57	0.38	5.79	7.35	7.39	0.49	6.38	8.40
OTSPSFC	10.28	1.10	8.02	12.55	12.10	0.73	10.60	13.59	12.33	0.94	10.40	14.26
DMSPC	91.32	2.62	85.93	96.70	91.83	1.73	88.27	95.39	89.01	2.23	84.41	93.60
DMSMDLAD	4,835.06	332.58	4,150.11	5,520.01	3,188.90	219.75	2,736.31	3,641.50	2,466.32	283.80	1,881.82	3,050.82
DMSPEGE	0.04	0.06	−0.09	0.17	0.04	0.04	−0.05	0.12	0.09	0.05	−0.02	0.20
RVPA	0.90	0.02	0.85	0.95	0.92	0.02	0.88	0.95	0.95	0.02	0.91	0.99
RVPMDL	657.81	68.07	517.61	798.01	494.93	44.98	402.29	587.57	421.81	58.09	302.17	541.45
SSTSSRT	256.98	14.51	227.09	286.86	239.52	9.59	219.77	259.27	218.15	12.38	192.64	243.65

Covariates appearing in the model are evaluated at the following values: Age = 41.59 years, BMI = 26.78 kg/m^2^.

RTIFDMRT, Median duration between stimulus onset and release of button; RTIFMMT, Mean time taken to touch stimulus after button release; SWMBE, Between errors—number of times that a box in which a token has been previously found is revisited; SWMS, Number of distinct boxes used for the subject to begin new search for a token; PRMPCI, Number of correct responses made in immediate condition; PRMPCD, Number of correct responses made in delayed condition; ERTOMDRT, Median latency of response from stimulus onset to subject response; ERTTH, Number of correctly answered responses; ASTSWMD, Median latency of response in rule switching trials; ASTLCMD, Median latency of response on congruent trials; SSPFSL, Longest sequence successfully recalled—forward variant; SSPRSL, Longest sequence successfully recalled—reversed variant; PALTEA, Number of times incorrect box is chosen + adjusted estimated number of errors that would have been made on any problems, attempts, and recalls that were not reached; PALFAMS, Number of correct box choices made on first attempt; DMSPC, Percentage of assessment trials during which subject selected the correct box on their first box choice; DMSMDLAD, Median latency from the available choices being displayed to the subject choosing the correct choice; DMSPEGE, Reports the probability of an error occurring when the previous trial was responded incorrectly; RVPMDL, Median response latency during assessment sequence blocks where the subject responded correctly; RVPA, Measure of how good the subject is at detecting target sequences; SSTSSRT, Length of time between the go stimulus and the stop stimulus at which the subject is able to successfully inhibit their response on 50% of the trials; OTSPSFC, Number of assessment problems on which the first box choice made was correct.

**Figure 1:**
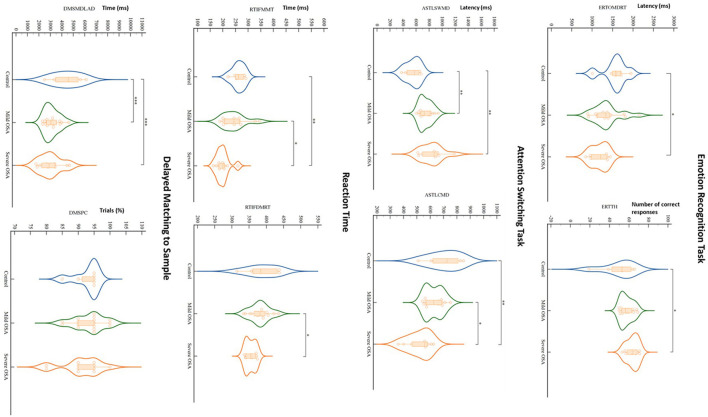
Violin plots depict results of significant CANTAB tests' findings for controls, mild OSA and severe OSA. When controlled for the influence of age and BMI, out of all CANTAB modalities (23 tests in 11 modalities), only Emotion Recognition Task, Attention Switching Task, Reaction Time and Delayed Matching to Sample showed significant differences between the groups, mainly between controls and severe OSA. Dots, squares and triangles show individual values (control, mild OSA and severe OSA, respectively). Thick dashed lines inside violins indicate group median, with thinner dashed lines indicating quartiles (*P < 0.05, **P < 0.01, ***P < 0.0001; two-way ANCOVA with Bonferroni's correction for multiple comparisons controlled for age and BMI). RTIFDMRT, Median duration between stimulus onset and release of button; RTIFMMT, Mean time taken to touch stimulus after button release; ERTOMDRT, Median latency of response from stimulus onset to subject response; ERTTH, Number of correctly answered responses; ASTSWMD, Median latency of response in rule switching trials; ASTLCMD, Median latency of response on congruent trials; DMSPC, Percentage of assessment trials during which subject selected the correct box on their first box choice; DMSMDLAD, Median latency from the available choices being displayed to the subject choosing the correct choice.

The cognitive findings for the whole set of behavioral readouts for the three experimental groups (control, mild OSA, and severe OSA), controlled for age and BMI and grouped into CANTAB sub-tests are shown in Table 3. Distinct deficits were observed in the tests investigating cognitive domains of vigilance, executive functioning, short-term visual recognition memory and social and emotion recognition, with the greatest number of differences between controls and those with severe OSA. Whilst subjects with mild OSA performed better than those with severe OSA on most of those same tasks, they were rarely worse than controls (see Figure 1).

**Table 3:** Summary of statistical analysis (two-way ANCOVA with Bonferroni's correction for multiple comparisons) for all CANTAB behavioral tasks and all pair-wise comparisons between groups (control, mild OSA, and severe OSA).

**Pairwise comparisons**	**Predicted (LS) mean diff**.	**95% CI of diff**.		**Below threshold**	**Summary**	**Adjusted *P*-value**	**Effect size**
		**Lower**	**Upper**				
**ASTLSWMD**							
Control vs. mild	−194.32	−345.94	−42.71	Yes	^**^	0.0090	−1.521
Control vs. severe	−224.73	−400.66	−48.80	Yes	^**^	0.0090	−1.698
Mild vs. severe	−30.41	−161.65	100.83	No	ns	1.0000	−0.230
**ASTLCMD**							
Control vs. mild	76.07	−39.03	191.17	No	ns	0.3070	0.784
Control vs. severe	182.28	48.71	315.84	Yes	^**^	0.0050	1.815
Mild vs. severe	182.28	6.57	205.84	Yes	^*^	0.0340	1.058
**ERTOMDRT**							
Control vs. mild	540.99	−72.07	1,154.05	No	ns	0.0970	1.047
Control vs. severe	806.60	95.22	1,517.98	Yes	^*^	0.0220	1.508
Mild vs. severe	265.61	−265.06	796.29	No	ns	0.6320	0.497
**ERTTH**							
Control vs. mild	−8.57	−21.01	3.87	No	ns	0.2680	−0.830
Control vs. severe	−15.96	−30.40	−1.53	Yes	^*^	0.0270	−1.470
Mild vs. severe	−7.39	−18.16	3.38	No	ns	0.2710	−0.681
**PALTEA**							
Control vs. mild	0.13	−13.12	13.39	No	ns	1.0000	0.012
Control vs. severe	5.52	−9.86	20.90	No	ns	1.0000	0.477
Mild vs. severe	5.39	−6.09	16.86	No	ns	0.7190	0.466
**PALFAMS**							
Control vs. mild	0.64	−4.03	5.32	No	ns	1.0000	0.163
Control vs. severe	−1.90	−7.33	3.53	No	ns	1.0000	−0.465
Mild vs. severe	−2.54	−6.59	1.51	No	ns	0.3590	−0.623
**PRMPCI**							
Control vs. mild	7.80	−2.08	17.67	No	ns	0.1610	0.937
Control vs. severe	3.19	−8.26	14.65	No	ns	0.5380	0.371
Mild vs. severe	−4.60	−13.15	3.95	No	ns	0.5380	−0.534
**PRMPCD**							
Control vs. mild	−10.22	−27.74	7.30	No	ns	0.4410	−0.692
Control vs. severe	−9.88	−30.21	10.44	No	ns	0.6710	−0.647
Mild vs. severe	0.33	−14.83	15.50	No	ns	1.0000	0.022
**RTIFMDRT**							
Control vs. mild	6.64	−33.59	46.87	No	ns	1.0000	0.196
Control vs. severe	42.25	−4.44	88.94	No	ns	0.0860	1.203
Mild vs. severe	35.61	0.78	70.44	Yes	^*^	0.0440	1.015
**RTIFMMT**							
Control vs. mild	26.05	−16.22	68.32	No	ns	0.3790	0.732
Control vs. severe	69.31	20.26	118.36	Yes	^**^	0.0040	1.879
Mild vs. severe	43.26	6.67	79.85	Yes	^*^	0.0170	1.173
**SWMBE**							
Control vs. mild	4.76	−9.68	19.20	No	ns	1.0000	1.443
Control vs. severe	9.23	−7.52	25.99	No	ns	1.0000	2.703
Mild vs. severe	4.47	−8.02	16.97	No	ns	1.0000	1.311
**SWMS**							
Control vs. mild	1.13	−2.79	5.04	No	ns	1.0000	0.797
Control vs. severe	0.08	−4.46	4.63	No	ns	1.0000	0.058
Mild vs. severe	−1.04	−4.43	2.35	No	ns	1.0000	−0.711
**SSPFSL**							
Control vs. mild	−1.10	−2.78	0.58	No	ns	0.3140	−0.726
Control vs. severe	−1.79	−3.74	0.16	No	ns	0.0790	−1.140
Mild vs. severe	−0.69	−2.14	0.76	No	ns	0.7030	−0.439
**SSPRSL**							
Control vs. mild	−0.66	−2.46	1.14	No	ns	1.0000	−0.227
Control vs. severe	−1.47	−3.56	0.61	No	ns	0.2460	−0.490
Mild vs. severe	−0.82	−2.37	0.74	No	ns	0.5740	−0.271
**OTSPSFC**							
Control vs. mild	−1.82	−5.27	1.63	No	ns	0.5660	−0.559
Control vs. severe	−2.05	−6.05	1.96	No	ns	0.6050	−0.615
Mild vs. severe	−0.23	−3.22	2.76	No	ns	1.0000	−0.125
**DMSPC**							
Control vs. mild	−0.52	−8.72	7.69	No	ns	1.0000	−0.075
Control vs. severe	2.31	−7.22	11.83	No	ns	1.0000	0.322
Mild vs. severe	2.82	−4.28	9.93	No	ns	0.9530	0.395
**DMSMDLAD**							
Control vs. mild	1,646.16	602.20	2,690.12	Yes	^***^	0.0009	1.872
Control vs. severe	2,368.74	1,157.36	3,580.13	Yes	^***^	0.0008	2.600
Mild vs. severe	722.59	−181.08	1,626.25	No	ns	0.1520	0.793
**DMSPEGE**							
Control vs. mild	0.00	−0.19	0.20	No	ns	1.0000	0.061
Control vs. severe	−0.05	−0.27	0.18	No	ns	1.0000	−2.809
Mild vs. severe	−0.05	−0.22	0.12	No	ns	1.0000	−2.869
**RVPA**							
Control vs. mild	−0.02	−0.09	0.06	No	ns	1.0000	−0.260
Control vs. severe	−0.05	−0.14	0.04	No	ns	0.5920	−0.693
Mild vs. severe	−0.03	−0.10	0.04	No	ns	0.7830	−0.442
**RVPMDL**							
Control vs. mild	162.88	−50.81	376.56	No	ns	0.1850	0.905
Control vs. severe	236.00	−11.96	483.96	No	ns	0.0660	1.265
Mild vs. severe	73.12	−111.85	258.10	No	ns	0.9600	0.392
**SSTSSRT**							
Control vs. mild	17.46	−28.09	63.01	No	ns	1.0000	0.455
Control vs. severe	38.83	−14.03	91.69	No	ns	0.2130	0.977
Mild vs. severe	21.37	−18.06	60.80	No	ns	0.5300	0.538

* *P* < 0.05.

** *P* < 0.01.

*** *P* < 0.001.

All values are evaluated at the following values: Age = 41.59 years, BMI = 26.78 kg/m^2^.

CANTAB tests (23 tests in 11 modalities) for controls, mild OSA and severe OSA. RTIFDMRT, Median duration between stimulus onset and release of button; RTIFMMT, Mean time taken to touch stimulus after button release; SWMBE, Between errors—number of times that a box in which a token has been previously found is revisited; SWMS, Number of distinct boxes used for the subject to begin new search for a token; PRMPCI, Number of correct responses made in immediate condition; PRMPCD, Number of correct responses made in delayed condition; ERTOMDRT, Median latency of response from stimulus onset to subject response; ERTTH, Number of correctly answered responses; ASTSWMD, Median latency of response in rule switching trials; ASTLCMD, Median latency of response on congruent trials; SSPFSL, Longest sequence successfully recalled—forward variant; SSPRSL, Longest sequence successfully recalled—reversed variant; PALTEA, Number of times incorrect box is chosen + adjusted estimated number of errors that would have been made on any problems, attempts, and recalls that were not reached; PALFAMS, Number of correct box choices made on first attempt; DMSPC, Percentage of assessment trials during which subject selected the correct box on their first box choice; DMSMDLAD, Median latency from the available choices being displayed to the subject choosing the correct choice; DMSPEGE, Reports the probability of an error occurring when the previous trial was responded incorrectly; RVPMDL, Median response latency during assessment sequence blocks where the subject responded correctly; RVPA, Measure of how good the subject is at detecting target sequences; SSTSSRT, Length of time between the go stimulus and the stop stimulus at which the subject is able to successfully inhibit their response on 50% of the trials; OTSPSFC, Number of assessment problems on which the first box choice made was correct; ns, non significant.

The most significant deficits, by comparison to the control group, were demonstrated in the tests that assess both simultaneous visual matching ability and short-term visual recognition memory for non-verbalizable patterns (Figure 1; DMS), tests of executive functioning and cued attentional set shifting (Figure 1; AST), in vigilance and psychomotor functioning (RTT), and lastly, in social cognition and emotion recognition (ERT).

For full details of the cognitive findings please refer to Tables 2, 3 and Supplementary material.

## 4. Discussion

We report a distinct pattern of circumscribed cognitive deficits in middle-aged male patients with severe OSA, in the absence of any overt neuropsychiatric, cardiovascular or metabolic co-morbidities (Bubu et al., 2020). The findings are largely in keeping with previous studies of OSA patients with associated multiple comorbidities that similarly showed aberrant executive-functioning, visuospatial short-term-memory, deficits in vigilance and psychomotor control (Bucks et al., 2017; Rosenzweig et al., 2022). Thus, arguably, our findings suggest that distinct OSA-driven processes, particularly when OSA is severe, may be sufficient for cognitive changes to occur as early as middle age, in otherwise healthy male individuals.

Remarkably, we also report, for the first time, diminished social cognition in this group of middle-aged severe OSA patients. Social and emotional cognition is an important ability to interpret and identify socially relevant information, known to be impaired in several psychiatric conditions, including major depressive disorder, and thought to be strongly associated with sleep physiology (Gujar et al., 2011; Weightman et al., 2014). In past studies, sleep deprivation has been shown to selectively impair the accurate judgment of human facial emotions, especially threat relevant and reward relevant categories (van der Helm et al., 2010). Significant deficits in emotional facial recognition have been previously also reported following a night of sleep fragmentation, without significant reduction of total sleep time (Lee et al., 2022). In keeping, it has been suggested that the disruption of normal sleep process, and not the reduction of sleep time, may likewise play the role (Lee et al., 2022). Moreover, there is evidence to suggest that emotional facial recognition can be sleep-stage dependent, with REM sleep known to play a critical role on both emotional and neutral face recognition (Cunningham and Payne, 2017; Lee et al., 2022). Thus, it is likely that sleep fragmentation and associated sleep loss in our OSA patients, particularly REM-related fragmentation (Cunningham and Payne, 2017), may act to impair discrete affective neural systems, disrupting the identification of salient affective social cues (van der Helm et al., 2010).

More recently, in a thought provoking set of studies, sleep loss and sleep's diminished quality and or quantity, which indeed present one of the important features of OSA, have also been linked to diminished altruism (Ben Simon et al., 2022). Specifically, the authors argued that sleep loss represents one previously unrecognized factor that may dictate whether humans choose to help each other, which they based on their observations at three different scales, within individuals, across individuals, and across societies (Ben Simon et al., 2022). For instance, in one of the studies, one night of sleep loss was shown to trigger the withdrawal of help from one individual to another, with the associated fMRI findings showing deactivation of key nodes within the social cognition brain network that facilitate prosociality (Ben Simon et al., 2022).

Following this argument and our findings, as well as taking into account that currently around one-seventh of the world's adult population, or approximately one billion people, are estimated to have OSA (Lyons et al., 2020), the clinical and societal impact of OSA's effects on cognition, even in the absence of any associated co-morbidities, dictates urgent attention and a joint multidisciplinary effort. It is increasingly evident that OSA's functional neuropsychiatric impact may go well beyond OSA's currently best recognized role in increasing driving and occupational accidents risks (Bucks et al., 2017; Rosenzweig et al., 2022).

We believe that our pilot study, despite limitations, including its size, a small control group, multiple comparisons, and a cross-sectional design, significantly contributes to understanding of the complex interplay between OSA-severity and cognitive problems. Critically, our data also reveal a threshold effect in the cognitive domain of executive functioning. Furthermore, it appears that cognitive deficits in this age group are greatest in male patients with severe OSA, likely suggestive of already existent widespread intricate physiologic central nervous changes, and in further support of early treatment for this patient group (Rosenzweig et al., 2015; Gnoni et al., 2021).

Finally, another important limitation to any direct translational generalization of our findings lies in inclusion of male participants only. Whilst this enabled controlling for possible effects of the oestrous cycle, it also prevents us from generalizing to female patients. Moreover, over the last decade, pioneering new findings suggest a spectrum of changes in the brain metabolism during the pre-, peri-, and post-menopausal period (Mosconi et al., 2021), all of which may arguably interplay with OSA pathomechanisms (Driver et al., 2005; Saaresranta et al., 2015), as well as underlie its links with neurodegenerative processes and cognitive deficits (Polsek et al., 2018) in female patients with OSA.

In conclusion, future multi-center multi-modal longitudinal studies should confirm these findings, as well as decipher how these cognitive deficits may interplay in men and women with other comorbidity-driven impairments over time.

## Data availability statement

The datasets presented in this article are not readily available because all data that support the findings of this study will be made available upon reasonable request from the corresponding author, once the appropriate ethics amendments are sought to accommodate this request. Requests to access the datasets should be directed to the U.K. Research Ethics Committee [Integrated Research Application System (IRAS)].

## Ethics statement

The studies involving human participants were reviewed and approved by the U.K. Research Ethics Committee [Integrated Research Application System (IRAS): IRAS-Project-ID-170912; REC-REF16/L0/0893]. The patients/participants provided their written informed consent to participate in this study.

## Author contributions

VG and IR designed the study. VG, DO'R, MJ, RB, and MM analyzed the data. All authors contributed equally to the writing and revision of this manuscript.

## References

[B1] Alchanatis M. Zias N. Deligiorgis N. Amfilochiou A. Dionellis G. Orphanidou D. (2005). Sleep apnea-related cognitive deficits and intelligence: an implication of cognitive reserve theory. J. Sleep Res. 14, 69–75. 10.1111/j.1365-2869.2004.00436.x 15743336

[B2] Aloia M. S. Arnedt J. T. Davis J. D. Riggs R. L. Byrd D. (2004). Neuropsychological sequelae of obstructive sleep apnea-hypopnea syndrome: a critical review. J. Int. Neuropsychol. Soc. 10, 772–785. 10.1017/S1355617704105134 15327723

[B3] American Academy of Sleep Medicine. (2014). The International Classification of Sleep Disorders - Third Edition *(ICS*D-3).

[B4] Ancoli-Israel S. Palmer B. W. Cooke J. R. Corey-Bloom J. Fiorentino L. Natarajan L.. (2008). Cognitive effects of treating obstructive sleep apnea in Alzheimer's disease: a randomized controlled study. J. Am. Geriatr. Soc. 56, 2076–2081. 10.1111/j.1532-5415.2008.01934.x 18795985 PMC2585146

[B5] Ben Simon E. Vallat R. Rossi A. Walker M. P. (2022). Sleep loss leads to the withdrawal of human helping across individuals, groups, and large-scale societies. PLoS Biol. 20, e3001733. 10.1371/journal.pbio.3001733 35998121 PMC9398015

[B6] Bench C. J. Frith C. D. Grasby P. M. Friston K. J. Paulesu E. Frackowiak R. S.. (1993). Investigations of the functional anatomy of attention using the stroop test. Neuropsychologia 31, 907–922. 10.1016/0028-3932(93)90147-R 8232848

[B7] Berry R. B. Brooks R. Gamaldo C. Harding S. M. Lloyd R. M. Quan S. F.. (2017). AASM scoring manual updates for 2017 (version 2.4). J. Clin. Sleep Med. 13, 665–666. 10.5664/jcsm.6576 28416048 PMC5406946

[B8] Bonsignore M. R. Baiamonte P. Mazzuca E. Castrogiovanni A. Marrone O. (2019). Obstructive sleep apnea and comorbidities: a dangerous liaison. Multidiscip. Respir. Med. 14, 8. 10.1186/s40248-019-0172-9 30809382 PMC6374907

[B9] Bubu O. M. Andrade A. G. Umasabor-Bubu O. Q. Hogan M. M. Turner A. D. de Leon M. J.. (2020). Obstructive sleep apnea, cognition and Alzheimer's disease: a systematic review integrating three decades of multidisciplinary research. Sleep Med. Rev. 50, 101250. 10.1016/j.smrv.2019.101250 31881487 PMC7593825

[B10] Bubu O. M. Kaur S. S. Mbah A. K. Umasabor-Bubu O. Q. Cejudo J. R. Debure L.. (2022). Obstructive sleep apnea and hypertension with longitudinal amyloid-beta burden and cognitive changes. Am. J. Respir. Crit. Care. Med. 206, 632–636. 10.1164/rccm.202201-0107LE 35550019 PMC9716897

[B11] Bucks R. S. Olaithe M. Eastwood P. (2013). Neurocognitive function in obstructive sleep apnoea: a meta-review. Respirology 18, 61–70. 10.1111/j.1440-1843.2012.02255.x 22913604

[B12] Bucks R. S. Olaithe M. Rosenzweig I. Morrell M. J. (2017). Reviewing the relationship between OSA and cognition: where do we go from here? Respirology 22, 1253–1261. 10.1111/resp.13140 28779504

[B13] Cambridge Cognition. (n.d.a). Pattern Recognition Memory (PRM). Available online at: https://www.cambridgecognition.com/cantab/cognitive-tests/memory/pattern-recognition-memory-prm/

[B14] Cambridge Cognition. (n.d.b). Spatial Working Memory (SWM). Available online at: https://www.cambridgecognition.com/cantab/cognitive-tests/executive-function/spatial-executive-function-swm/

[B15] Cambridge Cognition. (n.d.c). Multitasking Test. Available online at: https://www.cambridgecognition.com/cantab/cognitive-tests/executive-function/multitasking-test-mtt/

[B16] Cambridge Cognition. (n.d.d). Spatial Span (SSP). Available online at: https://www.cambridgecognition.com/cantab/cognitive-tests/memory/spatial-span-ssp/

[B17] Cambridge Cognition. (n.d.e). Paired Associates Learning (PAL).

[B18] Cambridge Cognition. (n.d.f). One Touch Stockings of Cambridge (OTS). Available online at: https://www.cambridgecognition.com/cantab/cognitive-tests/executive-function/one-touch-stockings-of-cambridge-ots/#:~:text=One%20Touch%20Stockings%20of%20Cambridge%20is%20a%20test%20of%20executive,and%20the%20working%20memory%20subdomains.&text=The%20participant%20is%20shown%20two%20displays%20containing%20three%20coloured%20balls

[B19] Cambridge Cognition. (n.d.g). Delayed Matching to Sample (DMS). Available online at: https://www.cambridgecognition.com/cantab/cognitive-tests/memory/delayed-matching-to-sample-dms/#:~:text=Delayed%20Matching%20to%20Sample%20assesses,%2C%20for%20non%2Dverbalisable%20patterns.&text=The%20participant%20is%20shown%20a,patterns%2C%20after%20a%20brief%20delay

[B20] Cambridge Cognition. (n.d.h). Stop Signal Task (SST). Available online at: https://www.cambridgecognition.com/cantab/cognitive-tests/executive-function/stop-signal-task-sst

[B21] Cooke J. R. Ayalon L. Palmer B. W. Loredo J. S. Corey-Bloom J. Natarajan L.. (2009). Sustained use of CPAP slows deterioration of cognition, sleep, and mood in patients with Alzheimer's disease and obstructive sleep apnea: a preliminary study. J. Clin. Sleep Med. 5, 305–309. 10.5664/jcsm.27538 19968005 PMC2725246

[B22] Cosentino F. I. I. Bosco P. Drago V. Prestianni G. Lanuzza B. Iero I.. (2008). The APOE ε4 allele increases the risk of impaired spatial working memory in obstructive sleep apnea. Sleep Med. 9, 831–839. 10.1016/j.sleep.2007.10.015 18083630

[B23] Coull J. T. Frackowiak R. S. J. Frith C. D. (1998). Monitoring for target objects: activation of right frontal and parietal cortices with increasing time on task. Neuropsychologia 36, 1325–1334. 10.1016/S0028-3932(98)00035-9 9863686

[B24] Cunningham T. J. Payne J. D. (2017). “Emotional memory consolidation during sleep,” in Cognitive Neuroscience of Memory Consolidation. eds N. Axmacher and B. Rasch (Cham: Springer), 133–159. 10.1007/978-3-319-45066-7_9

[B25] Driver H. S. McLean H. Kumar D. V. Farr N. Day A. G. Fitzpatrick M. F. (2005). The influence of the menstrual cycle on upper airway resistance and breathing during sleep. Sleep 28, 449–456. 10.1093/sleep/28.4.449 16171289

[B26] Eikermann M. Jordan A. S. Chamberlin N. L. Gautam S. Wellman A. Lo Y.-L.. (2007). The influence of aging on pharyngeal collapsibility during sleep. Chest 131, 1702–1709. 10.1378/chest.06-2653 17413053 PMC2278166

[B27] Ester E. F. Sprague T. C. Serences J. T. (2015). Parietal and frontal cortex encode stimulus-specific mnemonic representations during visual working memory. Neuron 87, 893–905. 10.1016/j.neuron.2015.07.013 26257053 PMC4545683

[B28] Esther Strauss E. M. S. S. (2006). *Otfried Spreen. A Compendium of Neuropsychological Tests: Administration, Norms, and Commentary*. Third Edn. Oxford: Oxford University Press.

[B29] Gagnon K. Baril A.-A. Gagnon J.-F. Fortin M. Décary A. Lafond C.. (2014). Cognitive impairment in obstructive sleep apnea. Pathol. Biol. 62, 233–240. 10.1016/j.patbio.2014.05.015 25070768

[B30] Glenthøj L. B. Albert N. Fagerlund B. Kristensen T. D. Wenneberg C. Hjorthøj C.. (2019). Emotion recognition latency, but not accuracy, relates to real life functioning in individuals at ultra-high risk for psychosis. Schizophr. Res. 210, 197–202. 10.1016/j.schres.2018.12.038 30595441

[B31] Gnoni V. Drakatos P. Higgins S. Duncan I. Wasserman D. Kabiljo R.. (2021). Cyclic alternating pattern in obstructive sleep apnea: a preliminary study. J. Sleep Res. 30, e13350. 10.1111/jsr.13350 33939202

[B32] Goldman-Rakic P. S. (1995). Cellular basis of working memory. Neuron 14, 477–485. 10.1016/0896-6273(95)90304-6 7695894

[B33] Gujar N. McDonald S. A. Nishida M. Walker M. P. (2011). A role for REM sleep in recalibrating the sensitivity of the human brain to specific emotions. Cereb. Cortex. 21, 115–123. 10.1093/cercor/bhq064 20421251 PMC3000566

[B34] Jones K. T. Berryhill M. E. (2012). Parietal contributions to visual working memory depend on task difficulty. Front. Psychiatry 3, 81. 10.3389/fpsyt.2012.00081 22973241 PMC3437464

[B35] Jordan A. S. McSharry D. G. Malhotra A. (2014). Adult obstructive sleep apnoea. Lancet 383, 736–747. 10.1016/S0140-6736(13)60734-5 23910433 PMC3909558

[B36] Keightley M. L. Chiew K. S. Anderson J. A. Grady C. L. (2011). Neural correlates of recognition memory for emotional faces and scenes. Soc. Cogn. Affect. Neurosci. 6, 24–37. 10.1093/scan/nsq003 20194514 PMC3023078

[B37] Kirkness J. P. Schwartz A. R. Schneider H. Punjabi N. M. Maly J. J. Laffan A. M.. (2008). Contribution of male sex, age, and obesity to mechanical instability of the upper airway during sleep. J. Appl. Physiol. 104, 1618–1624. 10.1152/japplphysiol.00045.2008 18420722 PMC2474771

[B38] Lavenex P. Suzuki W. A. Amaral D. G. (2002). Perirhinal and parahippocampal cortices of the macaque monkey: projections to the neocortex. J. Comp. Neurol. 447, 394–420. 10.1002/cne.10243 11992524

[B39] Lee A. C. H. Buckley M. J. Pegman S. J. Spiers H. Scahill V. L. Gaffan D.. (2005). Specialization in the medial temporal lobe for processing of objects and scenes. Hippocampus 15, 782–797. 10.1002/hipo.20101 16010661

[B40] Lee V. V. Schembri R. Jordan A. S. Jackson M. L. (2022). The independent effects of sleep deprivation and sleep fragmentation on processing of emotional information. Behav. Brain Res. 424, 113802. 10.1016/j.bbr.2022.113802 35181390

[B41] Levy P. Kohler M. McNicholas W. T. Barbé F. McEvoy R. D. Somers V. K.. (2015). Obstructive sleep apnoea syndrome. Nat. Rev. Dis. Primers 1, 15015. 10.1038/nrdp.2015.15 27188535

[B42] Lyons M. M. Bhatt N. Y. Pack A. I. Magalang U. J. (2020). Global burden of sleep-disordered breathing and its implications. Respirology 25, 690–702. 10.1111/resp.13838 32436658

[B43] Martin M. S. Sforza E. Roche F. Barthélémy J. C. Thomas-Anterion C. PROOF study group. (2015). Sleep breathing disorders and cognitive function in the elderly: an 8-year follow-up study the proof-synapse cohort. Sleep 38, 179–187. 10.5665/sleep.4392 25325480 PMC4288598

[B44] Mosconi L. Berti V. Dyke J. Schelbaum E. Jett S. Loughlin L.. (2021). Menopause impacts human brain structure, connectivity, energy metabolism, and amyloid-beta deposition. Sci. Rep. 11, 10867. 10.1038/s41598-021-90084-y 34108509 PMC8190071

[B45] Nikodemova M. Finn L. Mignot E. Salzieder N. Peppard P. E. (2013). Association of sleep disordered breathing and cognitive deficit in APOE epsilon4 carriers. Sleep 36, 873–880. 10.5665/sleep.2714 23729930 PMC3649829

[B46] Olaithe M. Bucks R. S. (2013). Executive dysfunction in OSA before and after treatment: a meta-analysis. Sleep 36, 1297–1305. 10.5665/sleep.2950 23997362 PMC3738038

[B47] Olaithe M. Bucks R. S. Hillman D. R. Eastwood P. R. (2018). Cognitive deficits in obstructive sleep apnea: insights from a meta-review and comparison with deficits observed in COPD, insomnia, and sleep deprivation. Sleep Med. Rev. 38, 39–49. 10.1016/j.smrv.2017.03.005 28760549

[B48] Olaithe M. Skinner T. C. Hillman D. Eastwood P. E. Bucks R. S. (2015). Cognition and nocturnal disturbance in OSA: the importance of accounting for age and premorbid intelligence. Sleep Breath 19, 221–230. 10.1007/s11325-014-1000-2 24839237

[B49] Osorio R. S. Gumb T. Pirraglia E. Varga A. W. Lu S.-E. Lim J.. (2015). Sleep-disordered breathing advances cognitive decline in the elderly. Neurology 84, 1964–1971. 10.1212/WNL.0000000000001566 25878183 PMC4433459

[B50] Pépin J.-L. Delavie N. Pin I. Deschaux C. Argod J. Bost M.. (2005). Pulse transit time improves detection of sleep respiratory events and microarousals in children. Chest 127, 722–730. 10.1378/chest.127.3.722 15764750

[B51] Pessoa L. McKenna M. Gutierrez E. Ungerleider L. G. (2002). Neural processing of emotional faces requires attention. Proc. Natl. Acad. Sci. U. S. A. 99, 11458–11463. 10.1073/pnas.172403899 12177449 PMC123278

[B52] Polsek D. Gildeh N. Cash D. Winsky-Sommerer R. Williams S. C. R. Turkheimer F.. (2018). Obstructive sleep apnoea and Alzheimer's disease: in search of shared pathomechanisms. Neurosci. Biobehav. Rev. 86, 142–149. 10.1016/j.neubiorev.2017.12.004 29223769 PMC6562163

[B53] Quan S. F. Wright R. Baldwin C. M. Kaemingk K. L. Goodwin J. L. Kuo T. F.. (2006). Obstructive sleep apnea-hypopnea and neurocognitive functioning in the Sleep Heart Health Study. Sleep Med. 7, 498–507. 10.1016/j.sleep.2006.02.005 16815753

[B54] Rosenzweig I. Glasser M. Crum W. R. Kempton M. J. Milosevic M. McMillan A.. (2016). Changes in neurocognitive architecture in patients with obstructive sleep apnea treated with continuous positive airway pressure. EBioMedicine 7, 221–229. 10.1016/j.ebiom.2016.03.020 27322475 PMC4909326

[B55] Rosenzweig I. Glasser M. Polsek D. Leschziner G. D. Williams S. C. R. Morrell M. J. (2015). Sleep apnoea and the brain: a complex relationship. Lancet Respir. Med. 3, 404–414. 10.1016/S2213-2600(15)00090-9 25887982

[B56] Rosenzweig I. Gosselin N. Bucks R. S. (2022). “Cognitive and neurologic aspects of obstructive sleep apnea,” in Encyclopedia of Respiratory Medicine, Vol. 17, 2nd Edn (Elseivier Doyma), 60–74. 10.1016/B978-0-08-102723-3.00127-X

[B57] Rosenzweig I. Weaver T. E. Morrell M. J. (2017). “Obstructive sleep apnea and the central nervous system,” in Principles and Practice of Sleep Medicine (Pleasanton, CA: Biashideng Publishing Group), 1154–1166.e1155. 10.1016/b978-0-323-24288-2.00117-3

[B58] Saaresranta T. Anttalainen U. Polo O. (2015). Sleep disordered breathing: is it different for females? ERJ Open Res. 1, 15. 10.1183/23120541.00063-2015 27730159 PMC5005124

[B59] Sarter M. Givens B. Bruno J. P. (2001). The cognitive neuroscience of sustained attention: where top-down meets bottom-up. Brain Res. Brain Res. Rev. 35, 146–160. 10.1016/S0165-0173(01)00044-3 11336780

[B60] Schembri R. Spong J. Graco M. Berlowitz D. J. COSAQ study team. (2017). Neuropsychological function in patients with acute tetraplegia and sleep disordered breathing. Sleep 40, zsw037. 10.1093/sleep/zsw037 28364492

[B61] Sforza E. Roche F. Thomas-Anterion C. Kerleroux J. Beauchet O. Celle S.. (2010). Cognitive function and sleep related breathing disorders in a healthy elderly population: the SYNAPSE study. Sleep 33, 515–521. 10.1093/sleep/33.4.515 20394321 PMC2849791

[B62] Todd J. J. Marois R. (2004). Capacity limit of visual short-term memory in human posterior parietal cortex. Nature 428, 751–754. 10.1038/nature02466 15085133

[B63] van der Helm E. Gujar N. Walker M. P. (2010). Sleep deprivation impairs the accurate recognition of human emotions. Sleep 33, 335–342. 10.1093/sleep/33.3.335 20337191 PMC2831427

[B64] Wallace A. Bucks R. S. (2013). Memory and obstructive sleep apnea: a meta-analysis. Sleep 36, 203–220. 10.5665/sleep.2374 23372268 PMC3543053

[B65] Walter J. Lee J. Y. Blake S. Kalluri L. Cziraky M. Stanek E.. (2023). A new wearable diagnostic home sleep testing platform: comparison with available systems and benefits of multi-night assessments. J. Clin. Sleep Med. 10.5664/jcsm.10432 36692166 PMC10152349

[B66] Weightman M. J. Air T. M. Baune B. T. (2014). A review of the role of social cognition in major depressive disorder. Front. Psychiatry 5, 179. 10.3389/fpsyt.2014.00179 25566100 PMC4263091

