# Supplementary material for: Distinct cognitive changes in male patients with obstructive sleep apnoea without co-morbidities
Source: Front Sleep. Author manuscript; Available in PMC 2024 Jan 11. (PMC7615516; doi:10.3389/frsle.2023.1097946)
Supplement: Supplement [file EMS193191-supplement-Supplement.pdf]

Supplement to: **Distinct Cognitive Changes in Male Patients with Obstructive Sleep Apnoea without Co-morbidities**

Methods and Materials

Preliminary analysis of cognitive parameters in patients with different OSA severities was undertaken as a part of the multimodal clinical study InCOSA (Clinical.Trials.Gov, 2020), approved by the Research-Ethics-Committee (IRAS-Project-ID-170912; REC-REF16/L0/0893). Twenty seven male adult (≥ 35 ≤ 70 years old)[1], non-obese (body mass index < 30 kg m^{-2}), mildly somnolent patients with a *de novo* diagnosis of OSA according to the ICSD criteria (International Classification of Sleep Disorders, III Edition; American Academy of Sleep Medicine, 2014)[2] and a group of healthy individuals who were initially age-, BMI- sex- and education-matched to OSA patients were identified, as previously described[3]. For the purpose of this preliminary analysis only those patients (n=27) and controls (n=7) who completed the Cambridge-Neuropsychological-Test-Automated-Battery (CANTAB), a validated, reliable, neuropsychological battery that consists of memory, learning, attention, problem solving, and executive function tests, were included in the analysis. Eleven cognitive domains, pre-selected[4] based on previous reports of OSA- and depression-related deficits, were tested via 23 different automated CANTAB tests.

The exclusion criteria stipulated no concomitant sleep disorders, neuropsychiatric or medical current or past history, no history of alcohol or (recreational) drug abuse, and no concurrent use of psychotropic drugs. Moreover, patients with predominant or exclusive rapid eye movement sleep-related OSA were excluded. The exclusion criteria also included smokers, professional drivers and shift workers[3].

Cambridge Neuropsychological Test Automated Battery (CANTAB)

The CANTAB is a highly sensitive, validated touchscreen-based cognitive assessment which tests multiple cognitive domains. The morning after the video polysomnography at around 8:30 all patients completed a cognitive test battery using an iPad supervised by a trained member of staff. A comprehensive and detailed description of all the tests used is available at https://www.cambridgecognition.com/cantab/

1. Reaction Time Task (RTT)

The RTT tests reaction time, movement time, and vigilance (Cambridge Cognition Limited, 2016), which are associated with motor pathway and right anterior hemispheric functioning (Coull, 1998). Participants hold down a button at the bottom of the screen until a yellow spot flashes in an empty circle at the top of the screen and the participant must touch the same empty circle as quickly as possible. Trials are performed with one (simple) and five empty circles (5-choice). Two outcome measures are the reaction time (median time of button release upon stimulus onset) and movement time (mean time taken to touch stimulus after button release) for the five-choice task.

2. Spatial Working Memory (SWM)

SWM tests the retention and manipulation of visuospatial data in non-verbal and visuospatial working memory, which are associated with frontal lobe function (Cambridge Cognition Limited, 2016). In this task, participants are asked to locate a 'token' by touching a box. In each trial, each box contains only one token (i.e. a token will never be found in the same box again). Across trials, the number of tokens and boxes increases from 3 to 6 to 9 to 12 . Outcomes are the number of times the participant touches a box that previously contained a token in trials with 4, 6, and 8 boxes.

3. Pattern Recognition Memory (PRM)

PRM tests short-term visual memory in a 2-choice forced discrimination paradigm in both immediate and delayed conditions (Cambridge Cognition Limited, 2016), which are associated

with frontoparietal and posterior parietal function (Todd and Marois, 2004; Pessoa et al., 2002). Participants are shown a series of visual patterns, with each pattern shown briefly in the middle of the screen. Participants then choose the original pattern when presented with a new distractor pattern (immediate). This is repeated for x target patterns. After a delay, participants are presented with target and new distractor pattern pairs and required to select the target. Outcomes are the number of correct patterns recognised in the immediate and delayed tasks.

4. *Emotion Recognition Task (ERT)*

The ERT assesses social cognition and emotion recognition[5] (Cambridge Cognition Limited, 2016), which are associated with the limbic system, inferior frontal gyrus, parietal lobe, cingulate cortex and inferior/middle temporal lobe functioning (Keightley et al., 2011). Participants are shown a computer-generated face for 200ms, after which the emotion displayed by the face must be selected from six options (sadness, happiness, fear, anger, disgust, and surprise). Outcomes are the median reaction time and the total number of correct answers. Those who suffer from depression provide more negative ratings of emotional expression, reflecting the well-known negative bias seen in depression, where positive or neutral stimuli are seen as more negative[6]. During testing, brief presentation encourages implicit processing, as opposed to conscious appraisal of the faces, and emotion recognition latency, rather than accuracy was reported in individuals at ultra-high risk of developing pyschosis[5].

5. *Attention Switching Task (AST)*

The AST (currently renamed by Cambridge Cognition as the ‘Multitasking Test’ [MTT]) tests executive functioning and cued attentional set shifting, which are functions of the medial frontal structures and the anterior right hemisphere (Cambridge Cognition Limited, 2016; Bench et al., 1993). In all three trials, an arrow, pointing either left or right, appears on the right

or left side (independent of arrow direction) of the screen. Additionally, the words ‘direction’ or ‘side’ appear at the top of the screen. In the first two trials, participants decide the direction in which the arrow is pointing or the side on which the arrow is located by pressing the left or right buttons on the bottom of the touchscreen. In the final trial, the two rulesets are mixed, and participants are required to adjust to changing ‘direction’ and ‘side’ rules. All the trials are preceded by a practice trial, which provides verbal feedback regarding their answers to ensure the participants understand the rules. The response latency for same-rule set and switching-rule set tasks are the outcome measures.

6. *Spatial Span Memory (SSP)*

SSP assesses visuospatial working memory which is associated with frontoparietal function[7,8]. Subjects are shown white boxes which briefly change colour in a variable sequence and participants must select the boxes in the order in which they were displayed or in the reverse order (for the backward variant). The outcome measures are the longest sequence successfully recalled, errors, number of attempts and speed of response.

7. *Paired Associates Learning (PAL)*

PAL tests episodic visuospatial memory and associative learning, which are mainly functions of the temporal lobe and a parahippocampal gyrus (Cambridge Cognition Limited, 2016). In this task, 12 boxes are located at the side of the screen. Different patterns with varying colours and shapes are briefly displayed in these boxes. The number of patterns increases from 2 to 4 to 8 to 12 as participants complete each level. Participants are asked to remember the locations of these patterns and touch the box corresponding to the pattern shown in the middle of the screen. If participants are incorrect, the patterns are shown again. The outcome measures are

the number of correct box choices on the first attempt (irrespective of the pattern count) and the adjusted total errors on the 12-pattern task.

8. One Touch Stockings of Cambridge (OTS)

OTS tests spatial planning and working memory (Cambridge Cognition Limited, 2016) and it is a measure of dorsolateral prefrontal cortex function[9]. Participants are shown two images of three coloured balls. The top image presents the arrangement of the balls that must be achieved through manipulating the bottom image by imaginarily moving the balls. The balls may be moved one at a time by touching the required ball, then touching the position to which it should be moved. The problems increase in complexity, from one move to four moves. The outcome measures include the number of problems solved on first choice, mean choices to correct, speed of response to first choice and mean latency to correct. Each of these measures may be calculated for all problems.

9. Delayed Matching to Sample (DMS)

DMS assesses both simultaneous visual matching ability and short-term visual recognition memory, for non-verbalizable patterns, which is associated with medial temporal lobe function[10,11] . Subjects are shown complex visual pattern, with varying shapes and colours, and are asked to match the pattern to one of four patterns that are displayed simultaneously or after a short delay of 0, 4, and 12 seconds. The outcome measures are the latency, the number of correct patterns selected and a statistical measure giving the probability of an error after a correct or incorrect response.

10. Rapid Visual Information Processing (RVP)

The RVP is a measure of sustained attention (Cambridge Cognition Limited, 2016), associated with frontoparietal function[12]. Participants are shown a white box in the middle of the screen which displays digits ranging from 2 to 9 at 100 digits per minute, in a pseudo-random order. Participants must detect target sequences (i.e. 2-5-8), and press a button at the centre of the screen in response. The outcome measures include latency, probability of false alarms and sensitivity.

11. Stop Signal Task (SST)

SST is a test of impulse control and response inhibition (Cambridge Cognition Limited, 2016 associated with prefrontal cortex function[12]. Subjects are shown an arrow which appears on screen facing either left or right and initially the participant learns to select the corresponding button on each side of the screen depending on the direction of the arrow. After this learning phase, the participant is introduced to a beep which signals to the participant not to make any response where the delay between the presentation of the arrow and the sound of the

beep is variable. The outcome measures are direction errors, proportion of successful stops, reaction time on Go trials, and stop signal reaction time.

References

1. Franssen T, Stijnen M, Hamers F, Schneider F. Age differences in demographic, social and health-related factors associated with loneliness across the adult life span (19–65 years): a cross-sectional study in the Netherlands. *BMC Public Health.* 2020;20(1):1118.
2. Medicine AAoS. The International Classification of Sleep Disorders - Third Edition (ICSD-3). . 2014.
3. Gnoni V, Drakatos P, Higgins S, et al. Cyclic alternating pattern in obstructive sleep apnea: A preliminary study. *J Sleep Res.* 2021;30(6):e13350.
4. Bucks RS, Olaithe M, Rosenzweig I, Morrell MJ. Reviewing the relationship between OSA and cognition: Where do we go from here? *Respirology.* 2017;22(7):1253-1261.
5. Glenthoj LB, Albert N, Fagerlund B, et al. Emotion recognition latency, but not accuracy, relates to real life functioning in individuals at ultra-high risk for psychosis. *Schizophr Res.* 2019;210:197-202.
6. Gollan JK, Pane HT, McCloskey MS, Coccaro EF. Identifying differences in biased affective information processing in major depression. *Psychiatry Res.* 2008;159(1-2):18-24.
7. Ester EF, Sprague TC, Serences JT. Parietal and Frontal Cortex Encode Stimulus-Specific Mnemonic Representations during Visual Working Memory. *Neuron.* 2015;87(4):893-905.
8. Jones KT, Berryhill ME. Parietal contributions to visual working memory depend on task difficulty. *Front Psychiatry.* 2012;3:81.
9. Goldman-Rakic PS. Cellular basis of working memory. *Neuron.* 1995;14(3):477-485.
10. Lavenex P, Suzuki WA, Amaral DG. Perirhinal and parahippocampal cortices of the macaque monkey: projections to the neocortex. *J Comp Neurol.* 2002;447(4):394-420.
11. Lee AC, Buckley MJ, Pegman SJ, et al. Specialization in the medial temporal lobe for processing of objects and scenes. *Hippocampus.* 2005;15(6):782-797.
12. Sarter M, Givens B, Bruno JP. The cognitive neuroscience of sustained attention: where top-down meets bottom-up. *Brain Res Brain Res Rev.* 2001;35(2):146-160.

General Linear Model

Notes

Output Created		14-FEB-2023 11:46:12
Comments		
Missing Value Handling	Definition of Missing	User-defined missing values are treated as missing.
	Cases Used	Statistics are based on all cases with valid data for all variables in the model.
Syntax		GLM ASTLSWMD ASTLCMD ERTOMDRT ERTTH PALTEA PALFAMS PRMPCI PRMPCD RTIFDMRT RTIFMMT SWMBE SWMS SSPSFSL SSPRSL OTSPSFC DMSPC DMSMDLAD DMSPEGE RVPA RVPMDL SSTSSRT BY Group WITH Age BMI /METHOD=SSTYPE(3) /INTERCEPT=INCLUDE /EMMEANS=TABLES(Group) WITH(Age=MEAN BMI=MEAN) COMPARE ADJ(BONFERRONI) /PRINT=DESCRIPTIVE ETASQ /CRITERIA=ALPHA(.05) /DESIGN=Age*BMI Group.
Resources	Processor Time	00:00:00,00
	Elapsed Time	00:00:00,00

Between-Subjects Factors

		Value Label	N
Group	1	Control	7
	2	Mild OSA	13
	3	Severe OSA	9

Descriptive Statistics

Group		Mean	Std. Deviation	N
ASTLSWMD	Control	553.0714	111.74019	7
	Mild OSA	760.5000	102.73408	13
	Severe OSA	797.4444	127.33260	9
	Total	721.8966	146.57143	29
ASTLCMD	Control	705.9286	121.86859	7
	Mild OSA	626.4231	65.12611	13
	Severe OSA	518.5000	78.30469	9
	Total	612.1207	108.92393	29
ERTOMDRT	Control	1855.2143	812.32479	7
	Mild OSA	1411.4615	333.63493	13
	Severe OSA	1194.3333	181.62926	9
	Total	1451.1897	511.15542	29
ERTTH	Control	50.1429	16.33431	7
	Mild OSA	57.3077	6.03303	13
	Severe OSA	64.0000	5.12348	9
	Total	57.6552	10.36228	29
PALTEA	Control	15.8571	11.59639	7
	Mild OSA	14.4615	9.50978	13
	Severe OSA	8.4444	8.98765	9
	Total	12.9310	10.01760	29
PALFAMS	Control	4.7143	3.72891	7
	Mild OSA	4.9231	3.70723	13
	Severe OSA	7.8889	3.14024	9
	Total	5.7931	3.70694	29
PRMPCI	Control	97.6200	4.06462	7
	Mild OSA	92.3085	9.89590	13
	Severe OSA	98.1478	5.55667	9
	Total	95.4028	7.90114	29
PRMPCD	Control	78.5729	12.59834	7
	Mild OSA	88.4623	12.97260	13
	Severe OSA	87.9633	13.24875	9
	Total	85.9203	13.19560	29
RTIFDMRT	Control	388.2143	45.25010	7
	Mild OSA	385.2692	28.04506	13
	Severe OSA	351.5000	14.30690	9
	Total	375.5000	33.22945	29
RTIFMMT	Control	262.4551	24.24309	7
	Mild OSA	241.5216	38.32100	13
	Severe OSA	200.8138	24.69532	9
	Total	233.9411	38.88736	29
SWMBE	Control	24.1429	11.61075	7
	Mild OSA	19.0000	8.72735	13
	Severe OSA	14.3333	12.51000	9
	Total	18.7931	10.94590	29
SWMS	Control	6.1429	2.41030	7
	Mild OSA	4.3846	3.15009	13
	Severe OSA	5.1111	3.01846	9
	Total	5.0345	2.93358	29
SSPSFSL	Control	5.7143	1.38013	7
	Mild OSA	6.9231	1.11516	13
	Severe OSA	7.6667	1.32288	9
	Total	6.8621	1.40723	29
SSPRSL	Control	6.1429	1.95180	7
	Mild OSA	6.5385	0.87706	13
	Severe OSA	7.2222	1.39443	9
	Total	6.6552	1.36998	29
OTSPSFC	Control	10.4286	4.27618	7
	Mild OSA	12.0769	1.70595	13
	Severe OSA	12.2222	1.78730	9
	Total	11.7241	2.57594	29
DMSPC	Control	90.7143	9.32227	7
	Mild OSA	91.9231	3.83974	13
	Severe OSA	89.4444	5.83333	9
	Total	90.8621	5.98644	29
DMSMDLAD	Control	4401.3571	1283.21948	7
	Mild OSA	3254.6538	664.07060	13
	Severe OSA	2781.1111	844.62976	9
	Total	3384.4828	1062.55855	29
DMSPEGE	Control	0.0286	0.07559	7
	Mild OSA	0.0385	0.13868	13
	Severe OSA	0.0926	0.18839	9
	Total	0.0529	0.14268	29
RVPA	Control	0.9049	0.09614	7
	Mild OSA	0.9183	0.04148	13

[illegible]	Severe OSA	0.9470	0.03579	9
	Total	0.9240	0.05797	29
RVPMDL	Control	617.2857	267.92456	7
	Mild OSA	501.0769	146.23723	13
	Severe OSA	451.2222	35.53177	9
	Total	513.6552	170.05651	29
SSTSSRT	Control	256.7857	54.63214	7
	Mild OSA	239.5469	28.94934	13
	Severe OSA	218.2867	14.16920	9
	Total	237.1100	35.63051	29

Multivariate Tests[a]

Effect		Value	F	Hypothesis df	Error df	Sig.	Partial Eta Squared
Intercept	Pillai's Trace	0.999	346,867[b]	21.000	5.000	0.000	0.999
	Wilks' Lambda	0.001	346,867[b]	21.000	5.000	0.000	0.999
	Hotelling's Trace	1456.841	346,867[b]	21.000	5.000	0.000	0.999
	Roy's Largest Root	1456.841	346,867[b]	21.000	5.000	0.000	0.999
Age * BMI	Pillai's Trace	0.760	,754[b]	21.000	5.000	0.709	0.760
	Wilks' Lambda	0.240	,754[b]	21.000	5.000	0.709	0.760
	Hotelling's Trace	3.166	,754[b]	21.000	5.000	0.709	0.760
	Roy's Largest Root	3.166	,754[b]	21.000	5.000	0.709	0.760
Group	Pillai's Trace	1.711	1.691	42.000	12.000	0.163	0.855
	Wilks' Lambda	0.016	1,662[b]	42.000	10.000	0.197	0.875
	Hotelling's Trace	16.411	1.563	42.000	8.000	0.260	0.891
	Roy's Largest Root	12.795	3,656[c]	21.000	6.000	0.057	0.928

a. Design: Intercept + Age * BMI + Group
b. Exact statistic
c. The statistic is an upper bound on F that yields a lower bound on the significance level.

Tests of Between-Subjects Effects

Source	Dependent Variable	Type III Sum of Squares	df	Mean Square	F	Sig.	Partial Eta Squared
Corrected Model	ASTLSWMD	273936,486[a]	3	91312.162	6.968	0.001	0.455
	ASTLCMD	143395,504[b]	3	47798.501	6.329	0.002	0.432
	ERTOMDRT	1959614,596[c]	3	653204.865	3.049	0.047	0.268
	ERTTH	801,180[d]	3	267.060	3.027	0.048	0.266
	PALTEA	305,773[e]	3	101.924	1.018	0.402	0.109
	PALFAMS	73,041[f]	3	24.347	1.953	0.147	0.190
	PRMPCI	358,947[g]	3	119.649	2.153	0.119	0.205
	PRMPCD	501,798[h]	3	167.266	0.956	0.429	0.103
	RTIFDMRT	7848,690[i]	3	2616.230	2.835	0.059	0.254
	RTIFMMT	16876,832[j]	3	5625.611	5.523	0.005	0.399
	SWMBE	383,071[k]	3	127.690	1.074	0.378	0.114
	SWMS	22,701[l]	3	7.567	0.867	0.471	0.094
	SSPSFSL	15,348[m]	3	5.116	3.190	0.041	0.277
	SSPRSL	6,396[n]	3	2.132	1.155	0.347	0.122
	OTSPSFC	16,209[o]	3	5.403	0.797	0.507	0.087
	DMSPC	43,181[p]	3	14.394	0.375	0.772	0.043
	DMSMDLAD	16081184,502[q]	3	5360394.834	8.628	0.000	0.509
	DMSPEGE	,024[r]	3	0.008	0.360	0.782	0.041
	RVPA	,008[s]	3	0.003	0.766	0.524	0.084
	RVPMDL	158996,213[t]	3	52998.738	2.036	0.134	0.196
	SSTSSRT	5977,048[u]	3	1992.349	1.684	0.196	0.168
Intercept	ASTLSWMD	812808.491	1	812808.491	62.029	0.000	0.713
	ASTLCMD	734730.969	1	734730.969	97.285	0.000	0.796
	ERTOMDRT	2542258.424	1	2542258.424	11.866	0.002	0.322
	ERTTH	7098.563	1	7098.563	80.469	0.000	0.763
	PALTEA	541.789	1	541.789	5.409	0.028	0.178
	PALFAMS	17.378	1	17.378	1.394	0.249	0.053
	PRMPCI	14381.603	1	14381.603	258.841	0.000	0.912
	PRMPCD	13795.153	1	13795.153	78.853	0.000	0.759
	RTIFDMRT	245182.781	1	245182.781	265.708	0.000	0.914
	RTIFMMT	88611.890	1	88611.890	86.992	0.000	0.777
	SWMBE	776.163	1	776.163	6.530	0.017	0.207
	SWMS	98.730	1	98.730	11.309	0.002	0.311
	SSPSFSL	76.570	1	76.570	47.737	0.000	0.656
	SSPRSL	104.649	1	104.649	56.683	0.000	0.694
	OTSPSFC	273.836	1	273.836	40.369	0.000	0.618
	DMSPC	14556.284	1	14556.284	378.965	0.000	0.938
	DMSMDLAD	6325841.000	1	6325841.000	10.182	0.004	0.289
	DMSPEGE	0.001	1	0.001	0.025	0.875	0.001
	RVPA	1.621	1	1.621	470.411	0.000	0.950
	RVPMDL	255367.833	1	255367.833	9.811	0.004	0.282
	SSTSSRT	104998.068	1	104998.068	88.771	0.000	0.780
Age * BMI	ASTLSWMD	3682.733	1	3682.733	0.281	0.601	0.011
	ASTLCMD	253.314	1	253.314	0.034	0.856	0.001
	ERTOMDRT	202668.549	1	202668.549	0.946	0.340	0.036
	ERTTH	42.255	1	42.255	0.479	0.495	0.019
	PALTEA	34.221	1	34.221	0.342	0.564	0.013
	PALFAMS	15.523	1	15.523	1.245	0.275	0.047
	PRMPCI	132.247	1	132.247	2.380	0.135	0.087
	PRMPCD	2.334	1	2.334	0.013	0.909	0.001
	RTIFDMRT	292.426	1	292.426	0.317	0.578	0.013
	RTIFMMT	561.705	1	561.705	0.551	0.465	0.022
	SWMBE	3.170	1	3.170	0.027	0.872	0.001
	SWMS	8.558	1	8.558	0.980	0.332	0.038
	SSPSFSL	0.252	1	0.252	0.157	0.695	0.006
	SSPRSL	1.488	1	1.488	0.806	0.378	0.031
	OTSPSFC	0.609	1	0.609	0.090	0.767	0.004
	DMSPC	10.307	1	10.307	0.268	0.609	0.011
	DMSMDLAD	5347311.699	1	5347311.699	8.607	0.007	0.256
	DMSPEGE	0.003	1	0.003	0.118	0.734	0.005
	RVPA	0.000	1	0.000	0.056	0.815	0.002
	RVPMDL	46683.568	1	46683.568	1.793	0.193	0.067
	SSTSSRT	1.049	1	1.049	0.001	0.976	0.000
Group	ASTLSWMD	167343.460	2	83671.730	6.385	0.006	0.338
	ASTLCMD	99935.849	2	49967.924	6.616	0.005	0.346
	ERTOMDRT	1841515.278	2	920757.639	4.298	0.025	0.256
	ERTTH	715.900	2	357.950	4.058	0.030	0.245
	PALTEA	155.278	2	77.639	0.775	0.471	0.058
	PALFAMS	32.362	2	16.181	1.298	0.291	0.094
	PRMPCI	276.726	2	138.363	2.490	0.103	0.166
	PRMPCD	407.722	2	203.861	1.165	0.328	0.085
	RTIFDMRT	7395.138	2	3697.569	4.007	0.031	0.243
	RTIFMMT	15008.646	2	7504.323	7.367	0.003	0.371
	SWMBE	241.319	2	120.660	1.015	0.377	0.075
	SWMS	8.277	2	4.139	0.474	0.628	0.037
	SSPSFSL	8.942	2	4.471	2.787	0.081	0.182

	SSPRSL	6.388	2	3.194	1.730	0.198	0.122
	OTSPSFC	14.267	2	7.133	1.052	0.364	0.078
	DMSPC	40.195	2	20.098	0.523	0.599	0.040
	DMSMDLAD	16055731.123	2	8027865.562	12.922	0.000	0.508
	DMSPEGE	0.012	2	0.006	0.284	0.755	0.022
	RVPA	0.007	2	0.003	1.008	0.379	0.075
	RVPMDL	158995.312	2	79497.656	3.054	0.065	0.196
	SSTSSRT	4424.348	2	2212.174	1.870	0.175	0.130
Error	ASTLSWMD	327592.703	25	13103.708			
	ASTLCMD	188808.324	25	7552.333			
	ERTOMDRT	5356221.611	25	214248.864			
	ERTTH	2205.372	25	88.215			
	PALTEA	2504.089	25	100.164			
	PALFAMS	311.718	25	12.469			
	PRMPCI	1389.038	25	55.562			
	PRMPCD	4373.672	25	174.947			
	RTIFDMRT	23068.810	25	922.752			
	RTIFMMT	25465.518	25	1018.621			
	SWMBE	2971.687	25	118.867			
	SWMS	218.264	25	8.731			
	SSPSFSL	40.100	25	1.604			
	SSPRSL	46.156	25	1.846			
	OTSPSFC	169.584	25	6.783			
	DMSPC	960.267	25	38.411			
	DMSMDLAD	15531674.240	25	621266.970			
	DMSPEGE	0.546	25	0.022			
	RVPA	0.086	25	0.003			
	RVPMDL	650741.839	25	26029.674			
	SSTSSRT	29569.873	25	1182.795			
Total	ASTLSWMD	15714433.500	29				
	ASTLCMD	11198264.250	29				
	ERTOMDRT	68388427.250	29				
	ERTTH	99406.000	29				
	PALTEA	7659.000	29				
	PALFAMS	1358.000	29				
	PRMPCI	265696.889	29				
	PRMPCD	218962.334	29				
	RTIFDMRT	4119924.750	29				
	RTIFMMT	1629466.498	29				
	SWMBE	13597.000	29				
	SWMS	976.000	29				
	SSPSFSL	1421.000	29				
	SSPRSL	1337.000	29				
	OTSPSFC	4172.000	29				
	DMSPC	240425.000	29				
	DMSMDLAD	363799841.500	29				
	DMSPEGE	0.651	29				
	RVPA	24.852	29				
	RVPMDL	8461145.500	29				
	SSTSSRT	1665960.332	29				
Corrected Total	ASTLSWMD	601529.190	28				
	ASTLCMD	332203.828	28				
	ERTOMDRT	7315836.207	28				
	ERTTH	3006.552	28				
	PALTEA	2809.862	28				
	PALFAMS	384.759	28				
	PRMPCI	1747.985	28				
	PRMPCD	4875.469	28				
	RTIFDMRT	30917.500	28				
	RTIFMMT	42342.350	28				
	SWMBE	3354.759	28				
	SWMS	240.966	28				
	SSPSFSL	55.448	28				
	SSPRSL	52.552	28				
	OTSPSFC	185.793	28				
	DMSPC	1003.448	28				
	DMSMDLAD	31612858.741	28				
	DMSPEGE	0.570	28				
	RVPA	0.094	28				
	RVPMDL	809738.052	28				
	SSTSSRT	35546.921	28				

a. R Squared = ,455 (Adjusted R Squared = ,390)
b. R Squared = ,432 (Adjusted R Squared = ,363)
c. R Squared = ,268 (Adjusted R Squared = ,180)
d. R Squared = ,266 (Adjusted R Squared = ,178)
e. R Squared = ,109 (Adjusted R Squared = ,002)
f. R Squared = ,190 (Adjusted R Squared = ,093)
g. R Squared = ,205 (Adjusted R Squared = ,110)
h. R Squared = ,103 (Adjusted R Squared = -,005)
i. R Squared = ,254 (Adjusted R Squared = ,164)
j. R Squared = ,399 (Adjusted R Squared = ,326)
k. R Squared = ,114 (Adjusted R Squared = ,008)
l. R Squared = ,094 (Adjusted R Squared = -,014)
m. R Squared = ,277 (Adjusted R Squared = ,190)
n. R Squared = ,122 (Adjusted R Squared = ,016)
o. R Squared = ,087 (Adjusted R Squared = -,022)
p. R Squared = ,043 (Adjusted R Squared = -,072)
q. R Squared = ,509 (Adjusted R Squared = ,450)
r. R Squared = ,041 (Adjusted R Squared = -,074)
s. R Squared = ,084 (Adjusted R Squared = -,026)
t. R Squared = ,196 (Adjusted R Squared = ,100)
u. R Squared = ,168 (Adjusted R Squared = ,068)

Estimated Marginal Means

Group

Estimates

Dependent Variable	Mean			Std. Error			95% Confidence Interval					
							Lower Bound			Upper Bound		
	Group			Group			Group			Group		
	Control	Mild OSA	Severe OSA	Control	Mild OSA	Severe OSA	Control	Mild OSA	Severe OSA	Control	Mild OSA	Severe OSA
ASTLSWMD	564,453[a]	758,774[a]	789,183[a]	48.300	31.915	41.217	464.977	693.044	704.296	663.929	824.505	874.070
ASTLCMD	702,944[a]	626,876[a]	520,667[a]	36.668	24.229	31.291	627.424	576.975	456.222	778.463	676.777	585.111
ERTOMDRT	1939,648[a]	1398,661[a]	1133,049[a]	195.303	129.050	166.661	1537.413	1132.878	789.804	2341.883	1664.444	1476.293
ERTTH	48,924[a]	57,493[a]	64,885[a]	3.963	2.619	3.382	40.762	52.099	57.920	57.086	62.886	71.850
PALTEA	14,760[a]	14,628[a]	9,241[a]	4.223	2.790	3.604	6.063	8.881	1.819	23.457	20.375	16.662
PALFAMS	5,453[a]	4,811[a]	7,353[a]	1.490	0.984	1.271	2.385	2.783	4.734	8.522	6.839	9.971
PRMPCI	99,777[a]	91,981[a]	96,582[a]	3.145	2.078	2.684	93.299	87.701	91.055	106.254	96.262	102.110
PRMPCD	78,286[a]	88,506[a]	88,171[a]	5.581	3.688	4.762	66.792	80.911	78.363	89.780	96.101	97.980

RTIFDMRT	391,422[a]	384,783[a]	349,172[a]	12.817	8.469	10.937	365.024	367.340	326.646	417.819	402.226	371.698
RTIFMMT	266,900[a]	240,848[a]	197,587[a]	13.467	8.898	11.492	239.165	222.521	173.920	294.635	259.174	221.255
SWMBE	23,809[a]	19,051[a]	14,576[a]	4.600	3.040	3.926	14.335	12.790	6.491	33.283	25.311	22.661
SWMS	5,594[a]	4,468[a]	5,509[a]	1.247	0.824	1.064	3.026	2.771	3.318	8.162	6.164	7.700
SSPSFSL	5,808[a]	6,909[a]	7,598[a]	0.534	0.353	0.456	4.708	6.182	6.659	6.909	7.636	8.538
SSPRSL	5,914[a]	6,573[a]	7,388[a]	0.573	0.379	0.489	4.733	5.793	6.381	7.095	7.353	8.396
OTSPSFC	10,282[a]	12,099[a]	12,328[a]	1.099	0.726	0.938	8.019	10.604	10.397	12.546	13.595	14.260
DMSPC	91,316[a]	91,832[a]	89,007[a]	2.615	1.728	2.232	85.931	88.273	84.412	96.702	95.391	93.603
DMSMDLAD	4835,060[a]	3188,903[a]	2466,317[a]	332.575	219.755	283.801	4150.109	2736.310	1881.819	5520.011	3641.496	3050.816
DMSPEGE	,038[a]	,037[a]	,086[a]	0.062	0.041	0.053	-0.090	-0.048	-0.024	0.167	0.122	0.195
RVPA	,902[a]	,919[a]	,949[a]	0.025	0.016	0.021	0.851	0.885	0.905	0.953	0.952	0.992
RVPMDL	657,809[a]	494,933[a]	421,809[a]	68.075	44.981	58.091	517.607	402.292	302.169	798.011	587.574	541.450
SSTSSRT	256,978[a]	239,518[a]	218,147[a]	14.511	9.589	12.383	227.091	219.770	192.644	286.864	259.266	243.651

a. Covariates appearing in the model are evaluated at the following values: Age = 41,5862, BMI = 26,7828.

Pairwise Comparisons

Dependent Variable	(I) Group	(J) Group	Mean Difference (I-J)	Std. Error	Sig.[b]	95% Confidence Interval for Difference[b]	
						Lower Bound	Upper Bound
ASTLSWMD	Control	Mild OSA	-194,321*	59.087	0.009	-345.936	-42.706
		Severe OSA	-224,730*	68.563	0.009	-400.660	-48.800
	Mild OSA	Control	194,321*	59.087	0.009	42.706	345.936
		Severe OSA	-30.409	51.146	1.000	-161.649	100.831
	Severe OSA	Control	224,730*	68.563	0.009	48.800	400.660
		Mild OSA	30.409	51.146	1.000	-100.831	161.649
ASTLCMD	Control	Mild OSA	76.068	44.857	0.307	-39.035	191.171
		Severe OSA	182,277*	52.051	0.005	48.715	315.839
	Mild OSA	Control	-76.068	44.857	0.307	-191.171	39.035
		Severe OSA	106,209*	38.829	0.034	6.574	205.843
	Severe OSA	Control	-182,277*	52.051	0.005	-315.839	-48.715
		Mild OSA	-106,209*	38.829	0.034	-205.843	-6.574
ERTOMDRT	Control	Mild OSA	540.987	238.919	0.097	-72.075	1154.049
		Severe OSA	806,600*	277.236	0.022	95.219	1517.981
	Mild OSA	Control	-540.987	238.919	0.097	-1154.049	72.075
		Severe OSA	265.612	206.812	0.632	-265.062	796.287
	Severe OSA	Control	-806,600*	277.236	0.022	-1517.981	-95.219
		Mild OSA	-265.612	206.812	0.632	-796.287	265.062
ERTTH	Control	Mild OSA	-8.569	4.848	0.268	-21.009	3.871
		Severe OSA	-15,961*	5.625	0.027	-30.396	-1.526
	Mild OSA	Control	8.569	4.848	0.268	-3.871	21.009
		Severe OSA	-7.392	4.196	0.271	-18.161	3.376
	Severe OSA	Control	15,961*	5.625	0.027	1.526	30.396
		Mild OSA	7.392	4.196	0.271	-3.376	18.161
PALTEA	Control	Mild OSA	0.132	5.166	1.000	-13.124	13.388
		Severe OSA	5.519	5.994	1.000	-9.862	20.901
	Mild OSA	Control	-0.132	5.166	1.000	-13.388	13.124
		Severe OSA	5.387	4.472	0.719	-6.087	16.861
	Severe OSA	Control	-5.519	5.994	1.000	-20.901	9.862
		Mild OSA	-5.387	4.472	0.719	-16.861	6.087
PALFAMS	Control	Mild OSA	0.642	1.823	1.000	-4.035	5.319
		Severe OSA	-1.899	2.115	1.000	-7.326	3.528
	Mild OSA	Control	-0.642	1.823	1.000	-5.319	4.035
		Severe OSA	-2.541	1.578	0.359	-6.590	1.507
	Severe OSA	Control	1.899	2.115	1.000	-3.528	7.326
		Mild OSA	2.541	1.578	0.359	-1.507	6.590
PRMPCI	Control	Mild OSA	7.795	3.848	0.161	-2.077	17.668
		Severe OSA	3.195	4.465	1.000	-8.261	14.650
	Mild OSA	Control	-7.795	3.848	0.161	-17.668	2.077
		Severe OSA	-4.601	3.330	0.538	-13.147	3.945
	Severe OSA	Control	-3.195	4.465	1.000	-14.650	8.261
		Mild OSA	4.601	3.330	0.538	-3.945	13.147
PRMPCD	Control	Mild OSA	-10.219	6.827	0.441	-27.738	7.299
		Severe OSA	-9.885	7.922	0.671	-30.213	10.443
	Mild OSA	Control	10.219	6.827	0.441	-7.299	27.738
		Severe OSA	0.334	5.910	1.000	-14.830	15.499
	Severe OSA	Control	9.885	7.922	0.671	-10.443	30.213
		Mild OSA	-0.334	5.910	1.000	-15.499	14.830
RTIFDMRT	Control	Mild OSA	6.639	15.680	1.000	-33.595	46.872
		Severe OSA	42.249	18.194	0.086	-4.436	88.935
	Mild OSA	Control	-6.639	15.680	1.000	-46.872	33.595
		Severe OSA	35,611*	13.572	0.044	0.784	70.438
	Severe OSA	Control	-42.249	18.194	0.086	-88.935	4.436
		Mild OSA	-35,611*	13.572	0.044	-70.438	-0.784
RTIFMMT	Control	Mild OSA	26.052	16.474	0.379	-16.219	68.324
		Severe OSA	69,313*	19.116	0.004	20.262	118.364
	Mild OSA	Control	-26.052	16.474	0.379	-68.324	16.219
		Severe OSA	43,260*	14.260	0.017	6.669	79.851
	Severe OSA	Control	-69,313*	19.116	0.004	-118.364	-20.262
		Mild OSA	-43,260*	14.260	0.017	-79.851	-6.669
SWMBE	Control	Mild OSA	4.758	5.628	1.000	-9.682	19.199
		Severe OSA	9.233	6.530	0.509	-7.523	25.989
	Mild OSA	Control	-4.758	5.628	1.000	-19.199	9.682
		Severe OSA	4.475	4.871	1.000	-8.025	16.975
	Severe OSA	Control	-9.233	6.530	0.509	-25.989	7.523
		Mild OSA	-4.475	4.871	1.000	-16.975	8.025
SWMS	Control	Mild OSA	1.126	1.525	1.000	-2.787	5.040
		Severe OSA	0.085	1.770	1.000	-4.456	4.626
	Mild OSA	Control	-1.126	1.525	1.000	-5.040	2.787
		Severe OSA	-1.042	1.320	1.000	-4.429	2.346
	Severe OSA	Control	-0.085	1.770	1.000	-4.626	4.456
		Mild OSA	1.042	1.320	1.000	-2.346	4.429
SSPSFSL	Control	Mild OSA	-1.100	0.654	0.314	-2.778	0.577
		Severe OSA	-1.790	0.759	0.079	-3.736	0.156
	Mild OSA	Control	1.100	0.654	0.314	-0.577	2.778
		Severe OSA	-0.690	0.566	0.703	-2.142	0.762
	Severe OSA	Control	1.790	0.759	0.079	-0.156	3.736
		Mild OSA	0.690	0.566	0.703	-0.762	2.142
SSPRSL	Control	Mild OSA	-0.659	0.701	1.000	-2.459	1.141
		Severe OSA	-1.474	0.814	0.246	-3.562	0.614
	Mild OSA	Control	0.659	0.701	1.000	-1.141	2.459
		Severe OSA	-0.815	0.607	0.574	-2.373	0.743
	Severe OSA	Control	1.474	0.814	0.246	-0.614	3.562
		Mild OSA	0.815	0.607	0.574	-0.743	2.373
OTSPSFC	Control	Mild OSA	-1.817	1.344	0.566	-5.266	1.633
		Severe OSA	-2.046	1.560	0.605	-6.049	1.957
	Mild OSA	Control	1.817	1.344	0.566	-1.633	5.266
		Severe OSA	-0.229	1.164	1.000	-3.215	2.757
	Severe OSA	Control	2.046	1.560	0.605	-1.957	6.049

	Severe OSA	Mild OSA	0.229	1.164	1.000	-2.757	3.215
DMSPC	Control	Mild OSA	-0.515	3.199	1.000	-8.724	7.693
		Severe OSA	2.309	3.712	1.000	-7.216	11.834
	Mild OSA	Control	0.515	3.199	1.000	-7.693	8.724
		Severe OSA	2.824	2.769	0.953	-4.281	9.930
	Severe OSA	Control	-2.309	3.712	1.000	-11.834	7.216
		Mild OSA	-2.824	2.769	0.953	-9.930	4.281
DMSMDLAD	Control	Mild OSA	1646,157*	406.847	0.001	602.196	2690.118
		Severe OSA	2368,743*	472.095	0.000	1157.358	3580.128
	Mild OSA	Control	-1646,157*	406.847	0.001	-2690.118	-602.196
		Severe OSA	722.585	352.172	0.152	-181.081	1626.252
	Severe OSA	Control	-2368,743*	472.095	0.000	-3580.128	-1157.358
		Mild OSA	-722.585	352.172	0.152	-1626.252	181.081
DMSPEGE	Control	Mild OSA	0.001	0.076	1.000	-0.195	0.197
		Severe OSA	-0.048	0.089	1.000	-0.275	0.180
	Mild OSA	Control	-0.001	0.076	1.000	-0.197	0.195
		Severe OSA	-0.049	0.066	1.000	-0.218	0.121
	Severe OSA	Control	0.048	0.089	1.000	-0.180	0.275
		Mild OSA	0.049	0.066	1.000	-0.121	0.218
RVPA	Control	Mild OSA	-0.016	0.030	1.000	-0.094	0.061
		Severe OSA	-0.047	0.035	0.592	-0.137	0.044
	Mild OSA	Control	0.016	0.030	1.000	-0.061	0.094
		Severe OSA	-0.030	0.026	0.783	-0.097	0.037
	Severe OSA	Control	0.047	0.035	0.592	-0.044	0.137
		Mild OSA	0.030	0.026	0.783	-0.037	0.097
RVPMDL	Control	Mild OSA	162.876	83.277	0.185	-50.812	376.563
		Severe OSA	236.000	96.633	0.066	-11.957	483.958
	Mild OSA	Control	-162.876	83.277	0.185	-376.563	50.812
		Severe OSA	73.124	72.086	0.960	-111.847	258.095
	Severe OSA	Control	-236.000	96.633	0.066	-483.958	11.957
		Mild OSA	-73.124	72.086	0.960	-258.095	111.847
SSTSSRT	Control	Mild OSA	17.460	17.752	1.000	-28.091	63.011
		Severe OSA	38.831	20.599	0.213	-14.026	91.687
	Mild OSA	Control	-17.460	17.752	1.000	-63.011	28.091
		Severe OSA	21.371	15.366	0.530	-18.059	60.800
	Severe OSA	Control	-38.831	20.599	0.213	-91.687	14.026
		Mild OSA	-21.371	15.366	0.530	-60.800	18.059

Based on estimated marginal means

*. The mean difference is significant at the ,05 level.

b. Adjustment for multiple comparisons: Bonferroni.

Multivariate Tests

	Value	F	Hypothesis df	Error df	Sig.	Partial Eta Squared
Pillai's trace	1.711	1.691	42.000	12.000	0.163	0.855
Wilks' lambda	0.016	1,662[a]	42.000	10.000	0.197	0.875
Hotelling's trace	16.411	1.563	42.000	8.000	0.260	0.891
Roy's largest root	12.795	3,656[b]	21.000	6.000	0.057	0.928

Each F tests the multivariate effect of Group. These tests are based on the linearly independent pairwise comparisons among the estimated marginal means.

a. Exact statistic

b. The statistic is an upper bound on F that yields a lower bound on the significance level.

Univariate Tests

Dependent Variable		Sum of Squares	df	Mean Square	F	Sig.	Partial Eta Squared
ASTLSWMD	Contrast	167343.460	2	83671.730	6.385	0.006	0.338
	Error	327592.703	25	13103.708			
ASTLCMD	Contrast	99935.849	2	49967.924	6.616	0.005	0.346
	Error	188808.324	25	7552.333			
ERTOMDRT	Contrast	1841515.278	2	920757.639	4.298	0.025	0.256
	Error	5356221.611	25	214248.864			
ERTTH	Contrast	715.900	2	357.950	4.058	0.030	0.245
	Error	2205.372	25	88.215			
PALTEA	Contrast	155.278	2	77.639	0.775	0.471	0.058
	Error	2504.089	25	100.164			
PALFAMS	Contrast	32.362	2	16.181	1.298	0.291	0.094
	Error	311.718	25	12.469			
PRMPCI	Contrast	276.726	2	138.363	2.490	0.103	0.166
	Error	1389.038	25	55.562			
PRMPCD	Contrast	407.722	2	203.861	1.165	0.328	0.085
	Error	4373.672	25	174.947			
RTIFDMRT	Contrast	7395.138	2	3697.569	4.007	0.031	0.243
	Error	23068.810	25	922.752			
RTIFMMT	Contrast	15008.646	2	7504.323	7.367	0.003	0.371
	Error	25465.518	25	1018.621			
SWMBE	Contrast	241.319	2	120.660	1.015	0.377	0.075
	Error	2971.687	25	118.867			
SWMS	Contrast	8.277	2	4.139	0.474	0.628	0.037
	Error	218.264	25	8.731			
SSPSFSL	Contrast	8.942	2	4.471	2.787	0.081	0.182
	Error	40.100	25	1.604			
SSPRSL	Contrast	6.388	2	3.194	1.730	0.198	0.122
	Error	46.156	25	1.846			
OTSPSFC	Contrast	14.267	2	7.133	1.052	0.364	0.078
	Error	169.584	25	6.783			
DMSPC	Contrast	40.195	2	20.098	0.523	0.599	0.040
	Error	960.267	25	38.411			
DMSMDLAD	Contrast	16055731.123	2	8027865.562	12.922	0.000	0.508
	Error	15531674.240	25	621266.970			
DMSPEGE	Contrast	0.012	2	0.006	0.284	0.755	0.022
	Error	0.546	25	0.022			
RVPA	Contrast	0.007	2	0.003	1.008	0.379	0.075
	Error	0.086	25	0.003			
RVPMDL	Contrast	158995.312	2	79497.656	3.054	0.065	0.196
	Error	650741.839	25	26029.674			
SSTSSRT	Contrast	4424.348	2	2212.174	1.870	0.175	0.130
	Error	29569.873	25	1182.795			

The F tests the effect of Group. This test is based on the linearly independent pairwise comparisons among the estimated marginal means.
